# Pubertal induction and transition to adult sex hormone replacement in patients with congenital pituitary or gonadal reproductive hormone deficiency: an Endo-ERN clinical practice guideline

**DOI:** 10.1530/EJE-22-0073

**Published:** 2022-03-29

**Authors:** A Nordenström, S F Ahmed, E van den Akker, J Blair, M Bonomi, C Brachet, L H A Broersen, H L Claahsen-van der Grinten, A B Dessens, A Gawlik, C H Gravholt, A Juul, C Krausz, T Raivio, A Smyth, P Touraine, D Vitali, O M Dekkers

**Affiliations:** 1 Pediatric Endocrinology, Department of Women’s and Children’s Health Karolinska Institutet, and Department of Pediatric Endocrinology and Inborn Errors of Metabolism, Astrid Lindgren Children’s Hospital, Karolinska University Hospital, Stockholm, Sweden; 2 Developmental Endocrinology Research Group, School of Medicine, Dentistry & Nursing, University of Glasgow, Royal Hospital for Children, Glasgow, UK; 3 Division of Pediatric Endocrinology and Obesity Center CGG, Department of Pediatrics, Erasmus MC Sophia Children’s Hospital, Erasmus University Medical Center, Rotterdam, The Netherlands; 4 Department of Endocrinology, Alder Hey Children’s Hospital, Liverpool, UK; 5 Department of Medical Biotechnology and Translational Medicine, University of Milan, Milan, Italy; 6 Department of Endocrine and Metabolic Diseases, IRCCS Istituto Auxologico Italiano, Milan, Italy; 7 Pediatric Endocrinology Unit, Hôpital Universitaire des Enfants HUDERF, Université Libre de Bruxelles, Bruxelles, Belgium; 8 Division of Endocrinology, Department of Medicine, Leiden University Medical Center, Leiden, The Netherlands; 9 Department of Pediatric Endocrinology, Amalia Childrens Hospital, Radboud University Medical Centre, Nijmegen, The Netherlands; 10 Department of Child and Adolescent Psychiatry and Psychology, Sophia Children’s Hospital Erasmus Medical Center, Rotterdam, Netherlands; 11 Department of Internal Medicine and Pediatrics, Faculty of Medicine and Health Sciences, University Ghent, Ghent, Belgium; 12 Department of Pediatrics and Pediatric Endocrinology, Faculty of Medical Sciences, Medical University of Silesia, Katowice, Poland; 13 Department of Endocrinology and Internal Medicine, Aarhus University Hospital, Aarhus, Denmark; 14 Department of Molecular Medicine, Aarhus University Hospital, Aarhus, Denmark; 15 Department of Growth and Reproduction, Copenhagen University Hospital – Rigshospitalet, Copenhagen, Denmark; 16 International Research and Research Training Centre for Endocrine Disruption in Male Reproduction and Child Health (EDMaRC) and Department of Clinical Medicine, University of Copenhagen, Copenhagen, Denmark; 17 Department of Biochemical, Experimental and Clinical Sciences ‘Mario Serio’, University of Florence, Florence, Italy; 18 New Children’s Hospital, Pediatric Research Center, Helsinki University Hospital, and Research Program Unit, Faculty of Medicine, Stem Cells and Metabolism Research Program, University of Helsinki, Helsinki, Finland; 19 Turner Syndrome Support Society in the UK, ePAG ENDO-ERN, UK; 20 Department of Endocrinology and Reproductive Medicine, Pitié Salpêtriere Hospital, Paris, France; 21 Sorbonne Université Médecine and Center for Endocrine Rare Disorders of Growth and Development and Center for Rare Gynecological Disorders, Paris, France; 22 SOD ITALIA APS – Italian Patient Organization for Septo Optic Dysplasia and Other Neuroendocrine Disorders – ePAG ENDO-ERN, Rome, Italy; 23 Department of Clinical Epidemiology, LUMC Leiden, Leiden, The Netherlands; 24 Department of Clinical Epidemiology, Aarhus University, Aarhus, Denmark

## Abstract

An Endo-European Reference Network guideline initiative was launched including 16 clinicians experienced in endocrinology, pediatric and adult and 2 patient representatives. The guideline was endorsed by the European Society for Pediatric Endocrinology, the European Society for Endocrinology and the European Academy of Andrology. The aim was to create practice guidelines for clinical assessment and puberty induction in individuals with congenital pituitary or gonadal hormone deficiency. A systematic literature search was conducted, and the evidence was graded according to the Grading of Recommendations, Assessment, Development and Evaluation system. If the evidence was insufficient or lacking, then the conclusions were based on expert opinion. The guideline includes recommendations for puberty induction with oestrogen or testosterone. Publications on the induction of puberty with follicle-stimulation hormone and human chorionic gonadotrophin in hypogonadotropic hypogonadism are reviewed. Specific issues in individuals with Klinefelter syndrome or androgen insensitivity syndrome are considered. The expert panel recommends that pubertal induction or sex hormone replacement to sustain puberty should be cared for by a multidisciplinary team. Children with a known condition should be followed from the age of 8 years for girls and 9 years for boys. Puberty induction should be individualised but considered at 11 years in girls and 12 years in boys. Psychological aspects of puberty and fertility issues are especially important to address in individuals with sex development disorders or congenital pituitary deficiencies. The transition of these young adults highlights the importance of a multidisciplinary approach, to discuss both medical issues and social and psychological issues that arise in the context of these chronic conditions.

## Introduction

### Puberty normal physiology

Puberty is the physiological process during which secondary sexual characteristics develop. The physical changes, driven by marked hormonal alterations, are not only limited to the development of secondary sexual characteristics but also include changes in body composition, brain, cardiovascular and skeletal development. In parallel, the adolescent matures psychosocially and emotionally. The onset of puberty depends on genetic, nutritional and environmental factors (1). Oestrogen and testosterone are produced and secreted in response to pituitary gonadotropins (luteinising hormone (LH) and follicle-stimulating hormone (FSH)), which are under the control of hypothalamic gonadotropin-releasing hormone (GnRH). There is a broad range for the age at pubertal onset.

In girls, the earliest visible sign of central puberty is usually breast budding, described as breast stage 2 (B2) on the Tanner scale (2). The average attainment age for breast stage 2 is less than 10 years and should appear before the age of 13 years (3, 4). Menarche usually occurs 2–3 years after the onset of puberty, after peak height velocity, which usually occurs between B3 and B4 (2, 5, 6). However, individual variation is large.

In boys, the first sign of pubertal development is an increase in testis volume, ≥4 mL assessed by orchidometer, which occurs at an average age of 11 years, and should appear before the age of 14 years. This is typically followed by the development of pubic and axillary hair (7). Peak height velocity usually coincides with a testis volume of about 10–12 mL (7). After this, voice breaking occurs (8).

### Mini-puberty

Mini-puberty is a period which describes the activation of the hypothalamic-pituitary-gonadal hormone axis (HPG axis) in the first months of life. The gonadotropin levels show a postnatal LH surge during the first 24 h followed by an increase at around 1 week and a peak between 1 and 3 months of age (9, 10). Compared to full-term infants, those born preterm or small for gestational age show an exaggerated postnatal HPG axis activity. In boys, HPG activation coincides with a testosterone peak, with penile and testicular growth and with final testicular descent if not already in the scrotum at birth. In girls, it coincides with an increase in oestradiol, infant breast development, uterine growth and maturation of ovarian follicles.

The complete biological significance of mini-puberty is still to be elucidated, but it affects brain development, may be responsible for differences in body composition and have long-term consequences for gonadal function (11, 12). After the age of 3 months, gonadotropins decrease towards the age of 6 months, although FSH levels in girls can remain elevated until 3–4 years of age. The infant activation of the HPG hormone axis is silenced from the age of 3–6 months until reactivation at the onset of puberty. Therefore, these first months of life provide a window of opportunity for investigating the HPG hormone axis (13, 14, 15). The analysis of basal gonadotropin and gonadal hormones at the age of 1–3 months of life is helpful when investigating infants with suspected central or primary hypogonadism.

### Deficiencies of sexual development; DSD and gonadotropin deficiency

Disorders or differences of sex development (DSD) is the umbrella term for congenital conditions associated with atypical chromosomal, gonadal or phenotypic sex development and can be divided into three main categories, that is, chromosomal DSD, 46,XY DSD and 46,XX DSD (16) (see Table 1). Chromosomal DSD with atypical sex chromosomes comprises conditions such as Turner syndrome (45,X and other karyotypic variants) in girls or Klinefelter syndrome (47,XXY) in boys. Children with mixed gonadal dysgenesis (MGD) conditions such as 45,X/46,XY mosaicism are brought up either as boys or girls, depending on several factors including the degree of prenatal masculinisation. In 46,XY DSD, there may be a problem in gonadal development, testosterone/DHT synthesis or action. It includes patients with complete gonadal dysgenesis, typically with a female phenotype at birth, no pubertal development and a uterus. It also includes patients with partial gonadal dysgenesis with varying degrees of masculinisation at birth as well as during puberty; some of their physical features overlap with those of patients with 46,XY partial androgen insensitivity (PAIS). 46,XY complete androgen insensitivity (CAIS) is associated with a female phenotype but without a uterus due to the production of anti-Müllerian hormone (AMH) by the testis during fetal development. 46,XY disorders of androgen synthesis are also associated with reduced masculinisation at birth, and, in some conditions such as 17β-hydroxysteroid dehydrogenase deficiency and 5α-reductase deficiency, the activation of isoenzymes in parallel to the increase of testosterone levels during puberty can result in spontaneous virilisation. The 46,XX DSD category includes the relatively frequent occurrence of patients with androgen excess due to congenital adrenal hyperplasia caused by 21-hydroxylase deficiency and also patients with 46,XX DSD with testis development due to, for example, the presence of sex-determining factor of Y (SRY) on one of the X chromosomes or on other chromosomes. Ovotesticular DSD is characterised by the presence of both testicular and ovarian tissue, with a karyotype that can be 46,XX, 46,XY or mosaic.

**Table 1:** Deficiencies of sexual development: DSD nomenclature and hypogonadotropic hypogonadism.

Sex chromosome DSD	46,XY DSD	46,XX DSD	Hypogonadotropic hypogonadism
(A) 45,X	(A) Disorders of gonadal (testicular) development	(A) Disorders of gonadal (ovarian) development	Congenital
Turner syndrome and variants	CGD or PGD Gonadal/testis regression Ovotesticular DSD Ovarian DSD	Gonadal dysgenesis Testicular DSD Ovotesticular DSD	Isolated CHH (normosmic or Kallmann sydrome) Syndromic CHH Multiple pituitary hormone deficiencies
(B) 47,XXY	(B1) Disorders of androgen synthesis	(B1) Androgen excess	Acquired
Klinefelter syndrome and variants	Androgen biosynthesis defect 17β-hydroxysteroid dehydrogenase, 5α-reductase 2 deficiency StAR mutations Cholesterol side-chain cleavage deficiency 17α-hydroxylase/17,20-lyase deficiency 3β-hydroxysteroid dehydrogenase 2 17β-hydroxysteroid dehydrogenase deficiency 5α-reductase 2 deficiency P450 oxidoreductase deficiency Leydig cell hypoplasia, aplasia LH receptor mutation Smith–Lemli–Opitz syndrome	21-hydroxylase deficiency 3β-hydroxysteroid dehydrogenase 2 11β-hydroxylase deficiency P450 oxidoreductase deficiency Glucocorticoid receptor mutations (*GR*) (19)	Cancer treatment sequelae Brain/pituitary tumour Inflammation Autoimmunity Trauma Functional hypothalamic amenorrhoea (caloric deficits, psychological distress) (20) Opioid-induced Metabolic disorders
	(B2) Disorders of androgen action	(B2) Fetoplacental	
	Androgen insensitivity syndrome due to AR mutations, CAIS,PAIS Drugs and environmental modulators	Aromatase deficiency P450 oxidoreductase deficiency	
(C) 45,X/46,XY, MGD	(C) Other	(C) Other	
	Persistent Müllerian duct syndrome Vanishing testis syndrome Syndromic associations Environmental endocrine disruptors	Müllerian agenesis/hypoplasia (MRKH) Vaginal atresia Uterine abnormalities Syndromic associations Environmental androgen exposure	
(D) 46,XX/46,XY			
chimeric, ovotesticular DSD			

AR, androgen receptor; CAIS, complete androgen insensitivity syndrome; CGD, complete gonadal dysgenesis; MGD, mixed gonadal dysgenesis; PAIS, partial androgen insensitivity syndrome; PGD, partial gonadal dysgenesis.

Individuals with DSD may be identified in the neonatal period because of atypical external genitalia, lack of neonatal minipuberty or may be diagnosed in puberty/adolescence when pubertal development is delayed, incomplete, absent or atypical (16). Girls may seek medical attention because of the absence of breast development and/or primary amenorrhoea or with increasing virilisation during puberty. Boys may present with short height, slow or non-progressing pubertal development and/or gynaecomastia. There is an increased risk of malignancy in patients with 46,XY DSD, especially if gonads are intra-abdominal, and patients may present with gonadal tumour-secreting steroid hormones, complicating the diagnosis and may even be the presenting symptom (17). In some cases, early gonadectomy may have been performed due to a high malignancy risk of the presence of a Y chromosome together with under-masculinisation (18).

Finally, deficiencies of sexual development requiring treatment also include patients with impaired gonadal function due to gonadotropin deficiency and congenital hypogonadotropic hypogonadism (CHH), which can be divided into normosmic CHH and Kallmann syndrome (CHH and deficient sense of smell). CHH can also be a part of multiple pituitary hormone deficiencies or part of a syndrome such as CHARGE or Waardenburg syndrome. Phenotypes associated with CHH apparently shortly after birth or during childhood are midline defects such as cleft lip and/or palate, dental anomaly, anomaly of digits, congenital hearing impairment, anosmia/hyposmia, microphallus/cryptorchisism and family history of CHH.

### The role of hormonal replacement therapy during puberty

It is not uncommon that testosterone or oestrogen replacement therapy (ERT) is required in adolescents with DSD or pituitary deficiency (see Table 2) (21). The overall aim of the therapy is to ensure that secondary sexual characteristics and maturation of the body and the brain occur at a similar pace to peers. This may also be the case even if puberty has started spontaneously but ceases to progress appropriately.

**Table 2:** Spontaneous puberty, sex hormone replacement and possibility of fertility in subjects with DSD conditions and hypogonadotropic hypogonadism.

	Spontaneous puberty	Sex hormone replacement	Fertility options	References
Dysgenetic gonads				
CGD, 46,XX and XY females	No	Yes	If uterus present, oocyte donation	22, 23, 24
PGD:males, 46,XY	57–85%	17–25%	Azoospermia microTESE and ICSI; oigozoospermia ICSI	
PGD: females, 46,XX	**Not reported**	Yes	If uterus present, oocyte donation	
MGD: males	EMS < 5, 63%EMS > 5, all	EMS < 5, allEMS >5, 25%	Azoospermic in 80%; microscopic focal spermatogenesis in 25%; ICSI; sperm donation	22, 25, 26
MGD: females	Possible	Yes	Uterus present, ART	
Turner syndrome	In 21–50% if mosaicism; menarche 15–30%	Yes Almost all	Uterus present, ART If mosaicism, spontaneous pregnancy possible, 7%	27, 28
Klinefelter syndrome	Yes, normal start of puberty. Regression of testis	Yes, usually late in puberty or after puberty	Azoospermia, micro_TESE sometimes possible due to areas of preserved spermatogenesis	29, 30, 31, 32
46,XX males	Yes; 13% with cryptorchidism or hypospadias	May need hormone replacement; >90% elevated FSH and LH1/3 have low testosterone	Sperm donation	33
OvoTestis	Possible if gonadal tissue is present	Depends on the presence of gonadal tissue	Uterus in 31% spontaneous pregnancy described if ovarian tissue and 46,XX	26, 34
Males				
Females				
46,XY DSD				
Steroid production	If gonads retained		Impaired spermatogenesis, TESE, ICSI ART	35, 36, 37
5αR	Yes	Common		
17β-HSD	Yes	Common		
CAIS	Yes	Yes	No	38
PAIS: males	EMS <5, 67%EMS >5, all	Yes, 83%	Azoo-oligozoospermia	
PAIS: females	Yes, virilising	Yes	No	
Hypopituitarism				
Isolated	Seldom, spontaneous puberty may be late	Yes FSH/LH	Possible with FSH/LH treatment	See Table 5
MPHD	Variable	Variable	Possible	
CDGP	Yes	No Initially at times	Yes	
MRKH	Yes	no	Uterus transplantation, research basis	39

CDGP, constitutional delay of growth and puberty; CGD, complete gonadal dysgenesis; EMS, external masculinisation score 1–10 (1 lowest, 10 highest) (40); MGD, mixed gonadal dysgenesis; MPHD, multiple pituitary hormone deficiency; MRKH, Mayer–Rockytansky–Kuster–Hauser syndrome, PGD, partial gonadal dysgenesis.

Regardless of the cause of hypogonadism, appropriate oestrogen or testosterone replacement will be required for puberty induction and puberty progression. This should mimic the physiological process inducing the secondary sexual characteristics, growth plate maturation and psychological functioning. In those with no identified cause of hypogonadism, the lack of pubertal features at the age of 13 years in girls and 14 years in boys should prompt investigations and may indicate the need for pharmacological puberty induction. In those with an identified cause of hypogonadism, puberty should be induced over a period of 2–4 years until a satisfactory outcome, usually when an adult dose has been reached. Sex steroid hormones are imperative for somatic and psychological wellbeing, also in the longer perspective, due to their effects on bone mineral density (BMD), haematopoiesis and cardiovascular, sexual and metabolic health.

### Aromatase inhibitors in puberty

Aromatase inhibitors inhibit the formation of oestrogens from androgens, leading to oestrogen depletion. Given that oestrogens mediate the growth spurt in both sexes and contribute to epiphyseal closure, it was hypothesised that oestrogen depletion would improve adult height in boys (41). Aromatase inhibitors have been given to prepubertal or pubertal boys for 1–2 years, to increase predicted adult height (42, 43). In girls, aromatase inhibitors have been used in combination with GnRH analogues attempting to increase adult height in early maturing girls (44) and in the experimental treatment of congenital adrenal hyperplasia (45). Aromatase inhibitors have been used in gynaecomastia, McCune–Albright syndrome, aromatase overexpression in patients with large calcifying Sertoli cell tumours and familial male-limited precocious puberty (46). The most common side effect is the loss of BMD.

In boys, aromatase inhibitors given prior to the onset of puberty do not seem to change the timing (47). In contrast, the situation is different once the central restraint of gonadotropin secretion is diminished at the onset of puberty when puberty is mainly controlled by the sex steroid-mediated feedback from the gonads. In both sexes, this feedback is mediated by oestrogen, and consequently, oestrogen depletion in boys at that time leads to increased gonadotropin and testosterone levels.

### Biochemical hormonal testing

The assessment of the HPG axis includes the quantification of serum concentrations of gonadotropins, FSH and LH, as well as gonadal sex steroids oestradiol (E2) and testosterone (T). In addition, gonadal peptides like inhibin B, AMH (48) and insulin-like factor 3 (49) may add useful information about the gonadal Sertoli and Leydig cell function, respectively. AMH is also used as a marker of ovarian reserve.

When puberty starts, the secretion of FSH and LH is very low and increases with pulsatility only at night time in peripubertal children. Ultrasensitive FSH and LH assays (i.e. detection limit < 0.1 IU/L) are required to separate prepubertal from pubertal children based on their serum concentrations (48). A basal LH concentration above 0.3 IU/L is considered evidence of pituitary activation, whereas a lower or even undetectable LH concentration does not exclude pubertal onset. Therefore, a short intravenous GnRH test may be needed, with a stimulated LH above 5 IU/L which is considered a pubertal response. Importantly, peak LH may reach values of almost 10 IU/L in 1–3 years old prepubertal children (50). The GnRH test is often used to diagnose central precocious puberty but has limited additional value in delayed puberty (51). Reference ranges covering all ages including the pubertal transition period are available for FSH and LH using ultrasensitive assays (52).

The quantification of E2 and T at low concentrations found in pre- and peripubertal boys and girls requires specific and sensitive assays. E2 quantification is needed in the evaluation of girls with premature thelarche, precocious and delayed puberty, boys and men with gynaecomastia, patients with suspected hypogonadism, as well as monitoring of hypogonadal girls during puberty induction with oestrogen. High accuracy, specificity and precision, as well as standardisation of E2 assays, are mandatory according to the Endocrine Society (53, 54), as well as to European guidelines (55). Some diurnal variation in E2 has been shown using an ultrasensitive immunoassay (56). Sensitive mass spectrometry-based methods, such as the liquid chromatography-tandem mass spectrometry (LC-MS/MS) methods, are now accepted as state-of-the-art methods for the quantitative analysis of T and E2 (57). Reference ranges for E2 and T determined by LC-MS/MS are available (58, 59).

### Puberty induction general issues to consider

#### Psychological function and social development

Puberty is associated with numerous physical, psychological and social changes. Recent functional MRI studies on brain development in adolescence elucidated the impact of steroids on neuropsychological maturation (60, 61). A better understanding of sex steroid action on the changes in cognitive, emotional and social functioning during the transition from childhood to adulthood has raised awareness that atypical pubertal development is not only harmful to physical maturation and health in general but also creates a delay in intellectual, emotional and social capacities. Therefore, the management of puberty and puberty induction should include the monitoring of psychosocial functioning.

#### Informing the child

The child needs to be informed about the medical condition in a continuous and age-appropriate process (62). By discussing how to inform the child already at an early stage, parents will be able to prepare themselves. Parents need to understand and be prepared for the physical, psychological and social changes during adolescence in order to become aware of the challenges their child will come across. Advisory booklets or informative websites on how to prepare and support their child will empower parents and make it easier for them to communicate with their child about medical condition and the challenges they may encounter (see websites (https://www.dsdfamilies.org/charity; https://www.dsdteens.org/; http://www.accordalliance.org/dsdguidelines/parents.pdf; https://www.fairview.org/patient-education/40119; https://www.connecticutchildrens.org/wp-content/uploads/2017/02/DSD_Resources.pdf)). In particular, for adolescents with DSD, timely information and sex education are important. Psychological counselling, dedicated educational websites and contacts with other patients may be helpful.

All DSD conditions *per se* may intensify parental focus on their child’s behaviour, particularly with respect to assigned gender at birth in children with DSD. Some parents seek reinforcement of the decisions made and may start worrying when they experience insufficient reassurance. In psychological counselling, information on psychological aspects of child development including play behaviour, development of gender preferences and development of knowledge on sex and gender will be helpful to take away the turmoil.

Importantly, gender role behaviour, interests and preferences are mostly neither possible nor desirable to change (63, 64, 65) and cannot be used to predict gender identity (66). Acceptance of behaviour is needed to enable the child to develop a positive self-esteem that is necessary to cope with the challenges that children with DSD will meet in puberty and adulthood. Children need support from their parents. Parents who feel shame, shyness or inability to cope or to protect will need support and reinforcement of their parental competency.

#### Gender development

Prenatal androgen exposure and action influence future gender role behaviour and interests. This has been shown in studies of patients with 46,XX CAH and in children with 46,XY DSD raised male or female with different levels of prenatal androgen exposure (67, 68, 69, 70). The influence of prenatal androgens on gender identity is less clear. It should be emphasised that gender role behaviour does not imply gender identity; follow-up studies indicate that, as adults, the vast majority of individuals with a DSD developed a gender identity in agreement with the gender assigned at birth (71, 72, 73, 74, 75, 76, 77, 78). Many individuals who changed gender may not only have been prenatally exposed to androgens but also in the first 6 months after birth (during the mini-puberty) and during puberty (73, 79, 80). Patients living in countries outside Europe and Northern America often have limited possibilities for medical evaluation and treatment, leading to a delay in clinical management (76, 81, 82). Reports on the psychosocial implications of this delay in clinical management indicate that patients face social stigmatisation (83, 84, 85). The assessment of gender development should be conducted before the hormonal induction of puberty.

#### Transition

For all patients with chronic disorders, the transfer to adult care represents a major change. In pediatric care, young people generally have a long-standing relationship and are comfortable with the team who is familiar with their illness and their personal and social history; however, consultations do not always meet the needs of the adolescents and their necessary empowerment (86, 87, 88). International recommendations have been developed to support a successful transition from paediatric to adult care (89, 90, 91). However, the majority of the recommendations focus on paediatric preparation and advocate for ongoing information about transition throughout the care pathway, the inclusion of the family, consideration of developmental aspects, patient education, coordination with primary care and adaptation of the timing of transfer to the individual’s situation. A prepared, coordinated transition has a positive impact on patients’ health, experience of care and use of care (92).

A transition program can be tailored to the individual’s needs and future clinical management and is preferably drafted in collaboration with the medical specialist who will become the patient’s doctor in adulthood (89, 90, 91, 93). The program should test the adolescent’s knowledge and skills, encourage the adolescent to discuss daily life challenges with the medical team and/or parents and challenge the adolescent to set goals for independence (94).

The transition of young adults with gonadal dysfunction, whatever the reason, illustrates the importance of a multidisciplinary approach, to discuss both medical issues (about hormonal replacement therapy, long-term consequences in terms of sexuality, fertility) and social and psychological issues that arise in the context of these chronic conditions (89, 95).

## Methods

### Guideline working group

These guidelines were developed on behalf of the European Reference Network on Rare Endocrine Conditions (Endo-ERN). The following societies were represented: the European Society of Endocrinology (ESE) (Dekkers), the European Society for Paediatric Endocrinology (ESPE) (Vd Akker and Gawlik), CH/SOD association (Vitali) and the Turner Syndrome Support Society (Smyth). The working group had one in-person meeting (December 2019) and one virtual meeting (June 2020). All participants completed conflict of interest forms.

A draft of the guideline was reviewed by five experts in the field (see ‘Acknowledgments’ section) and has been distributed to all Endo-ERN members for comments. In addition, the following societies and networks were asked to review the guidelines: ESPE, ESE and the European Academy of Andrology. Furthermore, patient representatives in the Endo-ERN were involved in the whole process.

### Target group and aims

This guideline was developed for health care providers who may see patients with DSD or hypogonadotropic hypogonadism (HH) in need of treatment to induce or sustain puberty. In general, these patients should preferably be treated by a multidisciplinary team of experts in an Endo-ERN Reference Centre and their affiliated regional healthcare providers. General practitioners and patients might also find the guideline useful. Additionally, the guideline can serve as a source document for the preparation of patient information leaflets and educational materials.

In clinical practice, when making treatment decisions, both the recommendations and the clinical judgement of the treating physician should be taken into account in a patient-centred, shared-decision process. Recommendations are not meant to replace clinical acumen. Certain recommendations may not be feasible in individual countries and must be interpreted in the context of available resources.

### Summary of methods used for guideline development

This guideline used Grading of Recommendations, Assessment, Development and Evaluation (GRADE) as a methodological base (96). The first step was to define clinical questions; the second step was to perform a systematic literature search. After including all relevant articles for each clinical question, we rated the quality of the evidence and estimated an average effect for specific outcomes if possible. The quality of the evidence behind the recommendations is classified as very low (+OOO), low (++OO), moderate (+++O) or strong (++++) per outcome (97). Formal evidence syntheses were performed and graded only for recommendations addressing our initial clinical questions. Not all recommendations were formally graded.

For the recommendations, we considered the quality of the evidence, the balance of desirable and undesirable outcomes and individual values and preferences (patient preferences, goals for health, costs, management inconvenience, feasibility of implementation, etc.) (98). The recommendations are worded as ‘recommend’ (strong recommendation) or ‘suggest’ (weak recommendation). The meaning of a strong recommendation is that all reasonably informed persons (clinicians, politicians and patients) would want the management in accordance with the recommendation. For a weak recommendation, most persons would still act in accordance with the guideline but a substantial number would not (99). Importantly, one cannot abstain from making recommendations when there is scarce evidence, as treatment decisions will have to be made anyway. Recommendations are accompanied by an explanation of why the recommendation was made.

### Clinical questions, eligibility criteria and endpoint definition

At the start of this guideline process, six clinical questions were formulated, for which we performed a systematic literature search and review (See Supplementary Appendix 1 for details, see section on supplementary materials given at the end of this article).

Question I: What is the optimal treatment to induce or sustain puberty in males with partial gonadal dysgenesis?
Question II: What is the optimal treatment to induce or sustain puberty in males with HH?
Question III: What is the optimal treatment to induce or sustain puberty in females with partial gonadal dysgenesis?
Question IV: What is the optimal treatment to induce or sustain puberty in females with HH?
Question V: What is the optimal treatment to induce or sustain puberty in patients with CAIS?
Question VI: What is the optimal treatment to induce or sustain puberty in patients with PAIS?

### Description of search and selection of literature

**Figure 1:**
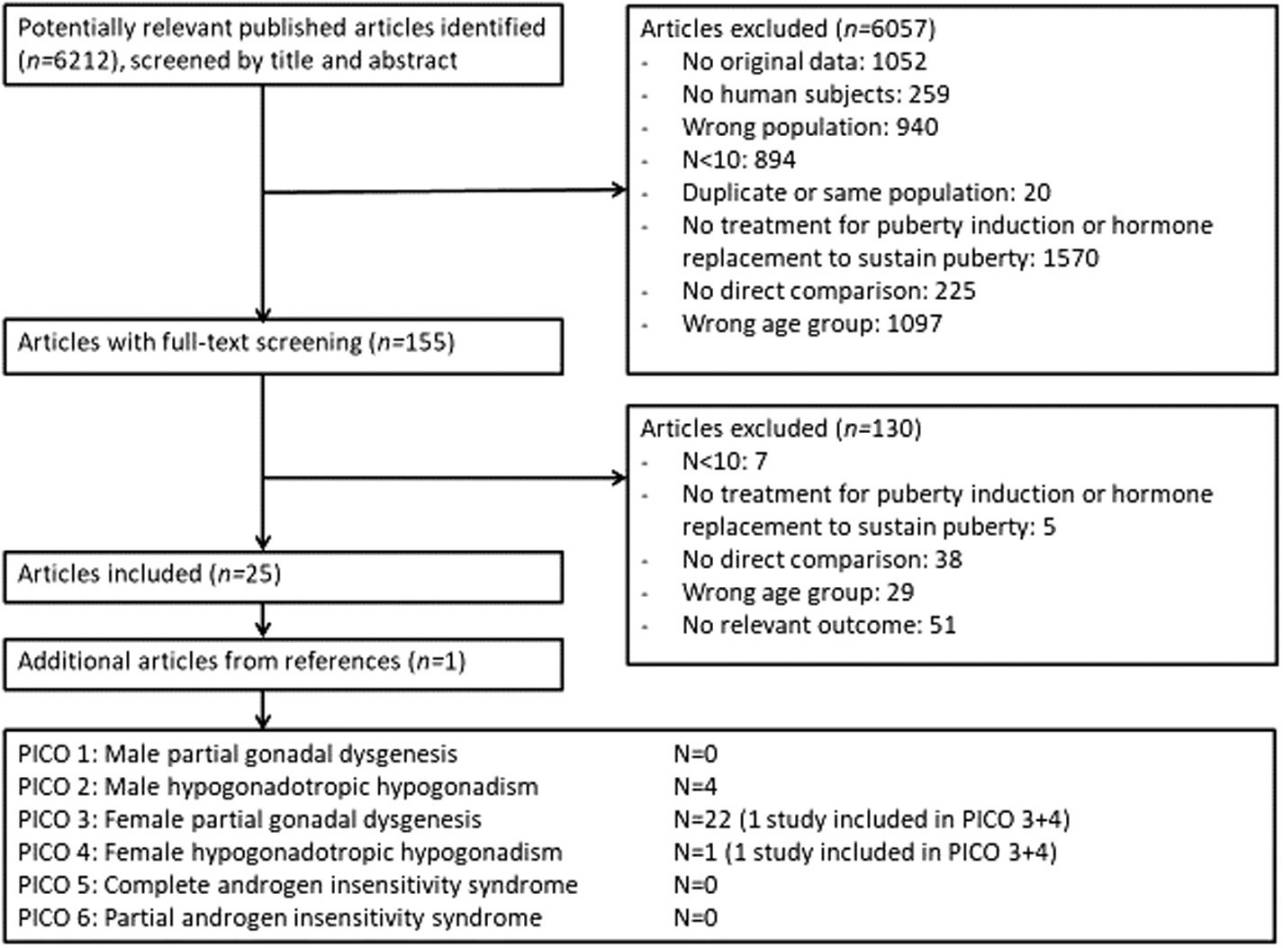
Flowchart of included papers.

We performed a literature search using five electronic medical databases in February 2020 (PubMed, Embase, Web of Science, COCHRANE and Emcare). No language restrictions were imposed. Due to similarities in treatments of interest and clinical outcomes, one overarching search strategy was used for all six clinical questions. References of included articles were checked to identify potentially relevant articles. Only articles studying a minimum of ten patients (to avoid including small studies with a high risk of selection bias), which directly compared at least two treatments to induce or sustain puberty (or one treatment vs placebo), were eligible for inclusion (Fig. 1).

In total, we identified 6212 papers with our search strategy. For questions I, V and VI (puberty treatment in male patients with partial gonadal dysgenesis, CAIS and partial androgen insensitivity syndrome), after a formal search and assessment of potentially relevant papers, no papers were found. For question II (puberty treatment in males with HH, we included four papers. For question III (puberty treatment in females with partial gonadal dysgenesis), we included 22 papers, of which 1 was also included for question IV. For question IV (puberty treatment in females with HH), we included one paper, which was also included for question III. A flow diagram of study inclusion is presented in Fig. 1.

## Summary and interpretation of the evidence from the systematic literature review

### Clinical question I What is the optimal treatment to induce or sustain puberty in boys with partial gonadal dysgenesis?

We found no studies on treatment to induce or sustain puberty in male patients with partial gonadal dysgenesis.

### Clinical question II What is the optimal treatment to induce or sustain puberty in boys with hypogonadotropic hypogonadism?

We included four studies on treatment to induce or sustain puberty in males with HH (100, 101, 102, 103), see Supplementary Appendix 2 Table 1 for the GRADE table and Supplementary Appendix 2 Table 2 for details of included studies and individual study outcomes. The four studies compared the use of i.m. testosterone vs no treatment (100), monthly i.m. testosterone vs weekly i.m. hCG (101), high- vs low-dose GnRH (102) and GnRH vs hCG (103). The following outcomes were investigated: Tanner stage, penile length, testicular volume, spermatogenesis, BMD, height, weight and BMI. Due to the small number of included studies, and the large variation in treatments used, no firm conclusion regarding optimal treatment to induce or sustain puberty in male HH can be drawn.

### Clinical question III What is the optimal treatment to induce or sustain puberty in female patients with partial gonadal dysgenesis?

We included 22 studies on treatment to induce or sustain puberty in female patients with partial gonadal dysgenesis, of which 1 was also included for clinical question IV; see Supplementary Appendix 3 Table 1 for the GRADE evidence table and Supplementary Appendix 3 Table 2 for study details including individual study outcomes. There were 10 randomised trials, 1 non-randomised trial and 11 cohort studies. In total, the studies included 1472 patients (there may be partial overlap in some study populations). Various oestrogen treatment regimens were compared: oestrogen vs no oestrogen, early (age 12–14 years) vs late (age 14–17 years) start of oestrogen, individualised vs fixed dose, oral vs transdermal administration and high vs low oestrogen dose.

**Figure 2:**
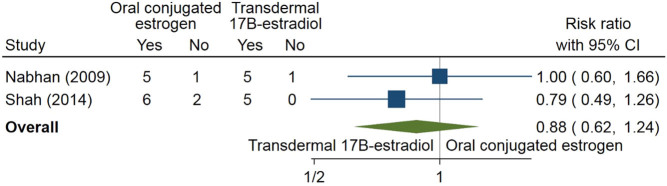
Oral-conjugated oestrogen vs transdermal 17β oestradiol for reaching Tanner stage B3. Yes denotes having reached Tanner stage 3; no denotes having not reached Tanner stage 3: the risk ratio expresses the probability ratio for reaching Tanner stage B3.

Eight different outcomes were studied: Tanner stage, menarche, uterine size, BMD, height, weight, BMI and liver function. In only four instances at least two studies describe the same outcome (e.g. height) presented in the same way (e.g. in cm) for the same comparison. In Fig. 2, the number of patients to reach Tanner stage B3 during the study period for oral conjugated oestrogen vs transdermal 17β oestradiol was meta-analysed for two studies (104, 105); no firm evidence for superiority, defined as patients reaching Tanner stage B3 or not, of one of the treatment modalities was found.

**Figure 3:**
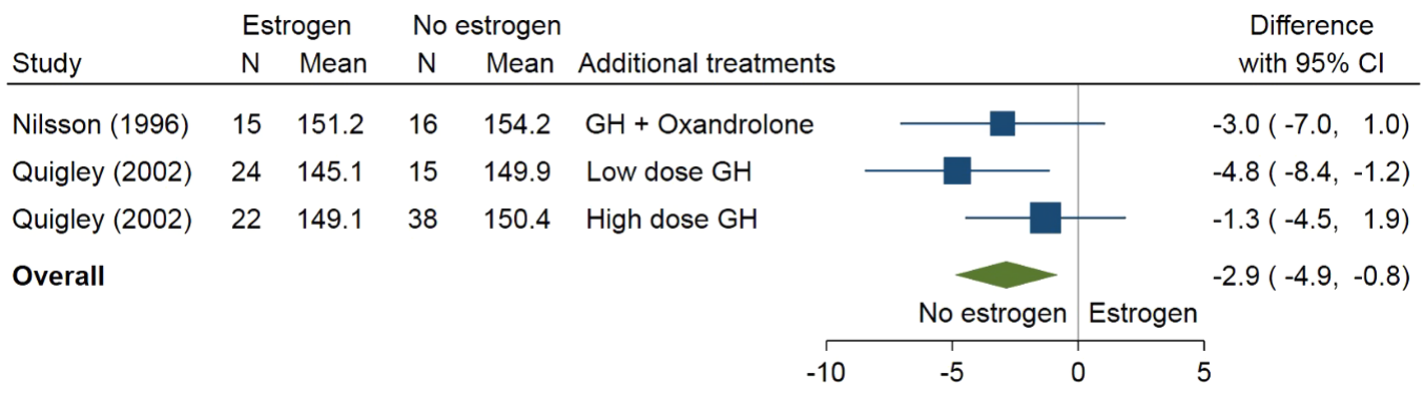
Oestrogen as add-on therapy for final height.

In Fig. 3, final height was compared, in a placebo-controlled study, between patients with Turner treated with oestrogen (ethinyl E2) vs patients, not on oestrogen in a meta-analysis using two studies (one split into patients on additionally a high or low dose of growth hormone) (106, 107). Patients on oestrogen had a lower final height than patients on placebo (difference −2.9 cm; 95% CI: −4.9 to −0.8 cm); it should be noted that these patients were treated with growth hormone.

**Figure 4:**
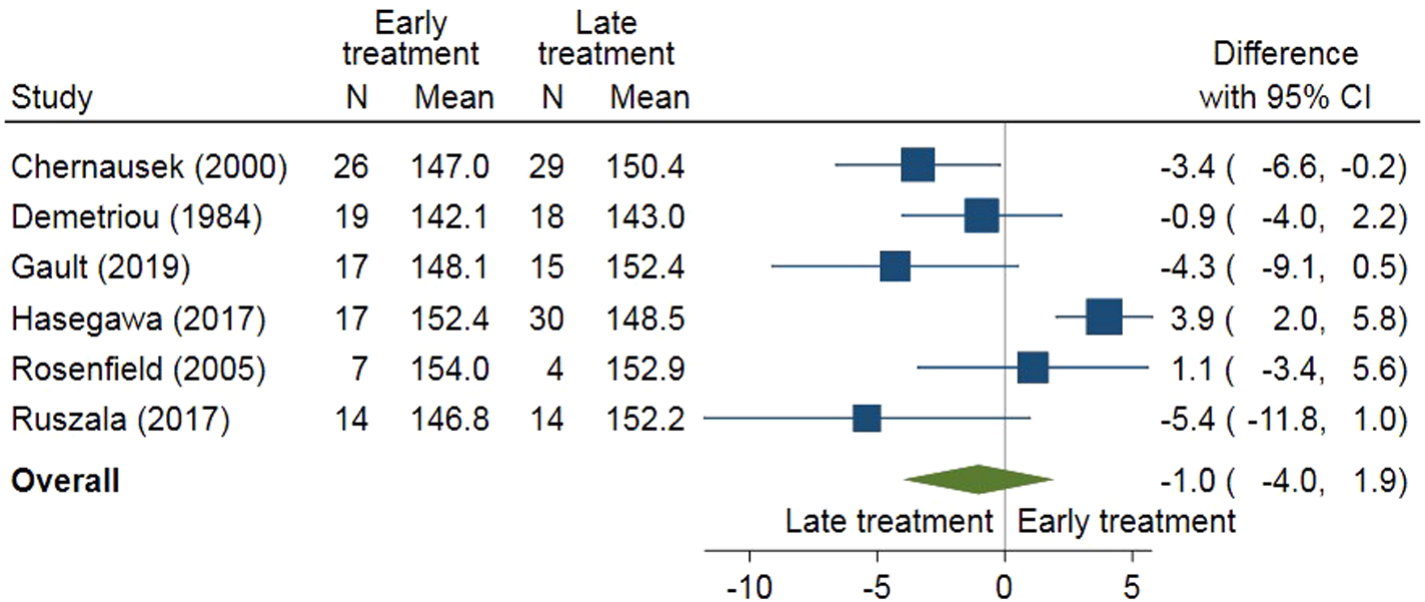
Early (age 10–12 years) and late (from age 12 years) start of oestrogen for final height.

In Fig. 4, final height was compared between early (age 10–12 years) and late (from age 12 years) start of oestrogen treatment (EE2 or E2) in a meta-analysis using six studies (108, 109, 110, 111, 112, 113). Early start of treatment did not result in a clearly lower final height than the late start of treatment (difference −1.0 cm; 95% CI: −4.0 to 1.9 cm).

**Figure 5:**
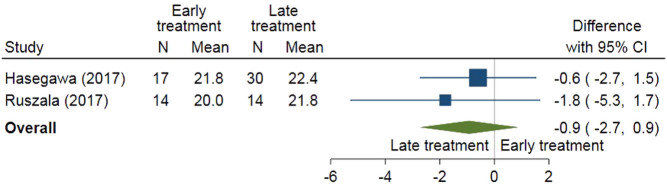
Early (age 10–12 years) and late (from age 12 years) start of oestrogen for BMI.

In Fig. 5, BMI was compared between early (age 10–12 years) and late (from age 12 years) start of oestrogen treatment in a meta-analysis using two studies (108, 111). The early start of treatment resulted in a lower, though not significantly, BMI than the late start of treatment (difference −0.9 points; 95% CI: −2.7 points to 0.9 points).

For all formal comparisons, the number of studies was low, with broad CIs and genuine uncertainty regarding the effect estimates.

### Clinical question IV What is the optimal treatment to induce or sustain puberty in female patients with hypogonadotropic hypogonadism?

We included 1 paper with 20 females with HH and treatment to induce or sustain puberty (105), which was also included for clinical question III and which did not present separate data for both patient categories. The GRADE evidence table is shown in Supplementary Appendix 4 Table 1, including details of the study and its outcomes. Patients used either oral 17β oestradiol, transdermal 17β oestradiol or oral-conjugated equine oestrogen. Patients using oral or transdermal 17β oestradiol more often reached Tanner breast stage 3 and had lower height and weight at the end of the study period than patients using oral-conjugated equine oestrogen. The number of patients is too small to draw firm conclusions on preferred treatment.

### Clinical question V What is the optimal treatment to induce or sustain puberty in women with complete androgen insensitivity syndrome?

We found no studies on treatment to induce or sustain puberty in patients with CAIS.

### Clinical question VI What is the optimal treatment to induce or sustain puberty in patients with partial androgen insensitivity syndrome?

We found no studies on treatment to induce or sustain puberty in patients with partial androgen insensitivity syndrome.

## Recommendations and rationales

### General

**R 1.1**We recommend that children and adolescents who need pubertal induction or sex hormone replacement to sustain puberty should be treated by a multidisciplinary team including a paediatric endocrinologist, adult endocrinologist, psychologist, urologist, gynaecologist, geneticist, surgeon and nurse specialist depending on the situation and specific requirements.

***Rationale***

Puberty and sexual maturation are sensitive and private matters and may be especially sensitive for individuals with a DSD. When the diagnosis is known since the neonatal period, the child and the parents can be informed and prepared well in advance of the time of puberty. If the diagnosis is made due to delayed, partial or absent pubertal development, the situation is more complex. In all cases, it is preferred that a specialised team with a multidisciplinary approach is involved (62).

The medical diagnosis requires patients and parents to cope with uncertainties regarding health and future development and to understand a complicated medical condition often associated with societal beliefs about gender and ‘normalcy’. As DSD conditions are rare, the expertise of medical professionals, also regarding many psychosocial aspects related to DSD, is essential. Discussing psychosocial aspects of DSD facilitates the acceptance of the condition and empowers parents to support their child whenever needed.

### Puberty induction in girls

#### Introduction

Accumulating data show that initiation of puberty at an age comparable with peers is essential for normal physiologic development, including secondary sex characteristics, bone, muscle, and social, sexual and psychologic development. Delayed pubertal induction, which is often the case in patients without pubertal development, may have longstanding consequences. An orderly and timely induction of puberty with the 17β-oestradiol in some form is the appropriate approach. The use of combined oral contraceptives to induce puberty is not recommended. However, there are currently still many uncertainties regarding the optimal pubertal induction regimen. Most of the recommendations below regarding oestrogen dosing are based on clinical experience and data in girls with Turner syndrome. Literature sources are scant regarding other types of female hypogonadism. Clinical experience in combination with the evidence from Turner syndrome data can, to some extent, be extrapolated to these groups.

#### In whom to consider puberty induction?

**R. 2.1**We recommend that an expert evaluation should be performed on any girl who does not show any sign of puberty by the age of 13 years, any girl who, by some definition, has delayed puberty (puberty s.d. score <−2) and all girls with a known condition/diagnosis that poses a risk of hypogonadism from the age of 8 years.

***Rationale***

Start of puberty in girls is typically defined by two signs of oestrogen action, the start of breast development, Tanner scale B2 and increased growth velocity. On average, this takes place at 10 years of age, the lower and upper limits of the normal range being after 8 years and before 13 years, respectively. Curve nomogram for pubertal progression exists (114):

Evaluation in girls with pubertal delay includes a detailed medical and family history. Laboratory investigations of gonadotropin levels and sex steroids will aid in differentiating between primary and HH. Primary gonadal failure such as complete or partial gonadal dysgenesis is confirmed by increased FSH and LH. Ultrasound or MRI should be performed to identify a uterus or Müllerian structures; however, a small uterus before oestrogen exposure may be missed by imagery. Bone age determination may be informative.

Karyotype or chromosomal array should be considered in all girls with delayed puberty and/or short stature to detect sex chromosomal abnormalities. The most common cause of primary ovarian failure is Turner syndrome due to 45,X karyotype or 45,X/46,XX or 45,X/46,XY mosaicism. These girls may have additional symptoms associated to the syndrome including cardiac anomalies. With the presence of Y chromosome material, there may be some degree of prenatal virilisation. Girls with complete gonadal dysgenesis can have 46,XY karyotype and a uterus (Swyer syndrome). Girls with ovo-testis may have 46,XX, 46,XY or mosaicism. Puberty may start spontaneously but come to a halt or be accompanied by virilisation due to the testicular component of the ovo-testis. The presence of Y material and under-masculinisation increases the risk of germ cell tumours.

The absence of menarche despite otherwise normal pubertal development should prompt a pelvic ultrasound to identify the uterus or Müllerian structures and to confirm or exclude Mayer–Rokitansky–Küster–Hauser syndrome or the Mullerian aplasia, renal anomalies, cervicothoracic somite dysplasia association (39).

Girls with 46,XY karyotype and deficiencies in testosterone or dihydrotestosterone synthesis virilise during puberty if their gonads have not been removed before puberty due to the activation of isoenzymes (21). They require a prompt diagnostic workup and psychological evaluation to discuss clinical management.

Girls with a known diagnosis predisposing or clearly resulting in primary gonadal failure or hypogonadal hypogonadism should be seen from the age of 8 years or earlier. This follow-up allows the DSD team to identify the individuals’ and family’s needs for clinical and psychological support throughout puberty and adolescence. This also allows time for the team to explain the diagnosis and prepare and inform the patient and parents about treatment decisions.

Girls with HH with low FSH, LH and anosmia such as in Kallmann syndrome or without anosmia require investigations to differentiate from constitutional delay or systemic diseases causing HPG axis suppression (115, 116).

**R. 2.2**We recommend to individualise but consider puberty induction at the age of 11 in most girls with gonadal dysgenesis or other syndromes with the absence of spontaneous puberty, who do not show signs of puberty and have confirmed hypogonadism after testing (++OO).

***Rationale***

Regardless of the cause, in female hypogonadism with insufficient pubertal progression, appropriate oestrogen replacement therapy (ERT) mimicking the physiology in timing and pace is the mainstay of puberty induction. The aim is to maintain the signs of puberty and obtain long-term effects by normalising uterine growth, attaining peak bone mass, influencing normal development of the brain and influencing metabolism, as well as sexual and psychological functioning. In the present context, we discuss conditions such as gonadal dysgenesis, HH (also transient forms), Turner syndrome, Prader Willi syndrome and others.

**R 2.3**We recommend that spontaneous puberty and treatable causes of hypogonadism should be ruled out before starting puberty induction in girls.

***Rationale***

The reasons for the lack of expected pubertal progression can be transient and/or need causal treatment. Differential diagnosis of female delayed puberty should take into account the constitutional delay of growth and puberty (CDGP) and maturational delay in the HPG axis secondary to an underlying non-reproductive condition. CDGP is responsible for a third of all cases of pubertal delay in teenage girls (117) and is mainly considered as part of the spectrum of normal puberty. To diagnose CDGP, congenital HH (CHH) needs to be ruled out. However, in some cases, the initial work-up fails to provide an unequivocal diagnosis. Current studies show that CDGP and CHH have distinct genetic profiles which may aid in discriminating between these conditions (118). Other causes of hypogonadism, as well as other pituitary deficiencies, should be treated but should not delay puberty induction.

**R. 2.4**We recommend to start individualised puberty induction in girls at the age of 11 years in cases where there are no signs of pubertal development and a diagnosis of hypogonadism is confirmed, or at the age of 13 years if the constitutional delay is suspected. (+OOO)

***Rationale***

ERT should be initiated around 11 years of age in cases with known hypogonadism which is slightly later than average. However, starting puberty induction at a younger age resulted in a lower final height (Fig. 4) and a lower BMI (Fig. 5) than starting puberty induction at a later age. The main goal of all therapeutic protocols for puberty induction in hypogonadism should be to achieve an endocrine milieu similar to natural processes with gradual increase of the oestrogen dose to mimic physiological pubertal development and obtain an appropriate adult phenotype with respect to uterine (when present), breast and bone development, body composition, as well as adult stature. In order to imitate the natural dynamic of puberty advancement, an incremental increase in the dose is recommended over a period of 2–3 years until satisfactory physical effect, usually an adult dose, has been reached (119, 120).

The age for the start of puberty treatment should be individualised. In individuals with tall stature and a tall final height prognosis, an earlier start of puberty treatment may be considered, for example in girls with 46,XY DSD. It may have to be delayed if CDGP is suspected and the results of a diagnostic work-up have to be awaited. In case of CDGP, treatment may be initiated as a short course of 3–6 months using low doses of oestradiol. Doses can be increased in order to mimic the normal course of puberty, but continuous monitoring for spontaneous resumption of progress and gonadotropin secretion is required.

The age of puberty induction also affects other aspects of life. Historically, much-delayed induction of puberty to hypothetically increase final height had long-lasting negative effects on sexual life in young adult women with Turner syndrome (TS) (121, 122).

**R 2.5**When premature ovarian insufficiency is seen, that is if appropriate progression fails, we recommend to start sex hormone replacement treatment also in girls who had a normal spontaneous start of puberty.

***Rationale***

Young women with spontaneous pubertal development and menstruations but markedly elevated gonadotropins need regular follow-up to start ERT before symptoms of premature ovarian insufficiency appear.

A considerable proportion of females with TS have spontaneous thelarche and/or menarche (20–40% show some degree of pubertal development, with menarche in approximately 16–20% and regular menstrual cycles in 6% of cases) (123, 124, 125, 126). A recent meta-analysis showed rates of menarche of 32 and 20% dependent on specific karyotype (127). Nevertheless, 80% or more of girls and women with TS require or will require ERT to initiate, progress or maintain pubertal development (128). As indicated by some observations, FSH level below 10 mIU/mL at 12 years and below 6.7 mIU/mL during mid-childhood (between 6 and 10 years) could be seen as an indicator of spontaneous puberty and the possibility of cyclical menstruation, but typically premature ovarian insufficiency within a few years will ensue (123, 129).

A similar situation may occur for females with mixed gonadal dysgenesis or partial gonadal dysgenesis. In females with ovo-testicular DSD and well-developed ovarian tissue, it often remains functional and menstruations have been reported to occur in 50% of cases (26). In individuals with spontaneous puberty and risk of premature ovarian failure, it is important to follow-up pubertal progression and measure gonadotropins in order to estimate ovarian reserve and assess the need for oestrogen replacement. AMH could be used as an additional laboratory marker. Values of AMH below –2 s.d. (4 pmol/L) predicted failure to enter puberty (130).

Inhibin B measurements have also been used to assess ovarian reserve (130, 131, 132). Undetectable inhibin B levels measured prior to pubertal onset were found in all patients with Turner syndrome and premature ovarian failure (133). However, due to its limited specificity (more than 1/3 of healthy girls have undetectable inhibin B levels during the menstrual cycle (follicular phase)), using inhibin B as a screening test to assess longitudinal ovarian reserve raises concerns (134). AMH appears to be a more reliable marker in this respect.

In case of premature ovarian insufficiency, decision regarding the dosing and the type and route of hormonal replacement therapy should be based on patient’s pubertal stage and the aim to mimic physiology.

#### Treatment approach and monitoring

**R 2.6**We recommend to use 17β-oestradiol for puberty induction or to sustain puberty in girls (++OO).

***Rationale***

Different types of sex hormones are used for ERT (see Table 3 for details). Especially for pubertal induction, bioidentical human oestrogens (oestradiol/17β-oestradiol E2) are the dominant formulations used presently and are preferred to non-bioidentical (e.g. ethinyl E2, conjugated equine oestrogens, dienestrol, and mestranol), synthetic or derived from animal sources. We acknowledge that different formulations may not be available in all countries.

**Table 3:** Oestrogen and progesterone preparations that can be used for pubertal induction and replacement therapy. This s not meant to be exhaustive, multiple preparations and brand names are available throughout the world.

Preparation	Doses available	Starting dose of puberty	Increase approximately every 6 months^*^ to adult dosing	Considerations for use
E_2_: transdermal options TD (some brands examples)		3–7 µg/day	25–100 µg/day	See text on applying patches
Menostar	14 µg	Part of patch twice weekly	Only used for low dosing situations, not fully hypogonadal replacement	The easiest way to give a low dose
Vivelle Dot	25, 37.5, 50, 75, 100 µg	Part of patch twice weekly or 1 patch per month (no patch for 3 weeks)^**^	25–100 µg twice weekly	Designed for twice weekly but can be given once per week to increase the dose slower
Vivelle Mini	25, 37.5, 50, 75, 100 µg	Part of patch twice weekly or 1 patch per month (no patch for 3 weeks)^**^	25–100 µg twice weekly	Smaller size patch, but not smaller dosing
Generic (different brands in different countries; e.g. Oesclim Estradot, Evorel, Systen, Climara, Demestril)	25, 37.5, 50, 75, 100 µg	Part of patch twice weekly or 1 patch per month (no patch for 3 weeks)^**^	25–100 µg twice weekly	Estradot: too small to properly cut into low doses and not stable in elevated temperature
Estraderm	25, 50, 100 µg	Part of patch twice weekly or 1 patch per month (no patch for 3 weeks)^**^	25–100 µg twice weekly	Reservoir form cannot be used to initiate puberty
Estraderm MX				
Divigel 0.1%	0.5 and 1.0 mg E_2_/sachet	Too potent for pubertal initiation	1–2 sachets daily	Cannot use to initiate puberty
Estragel 0.06%	0.75 mg E_2_/pump	Too potent for pubertal initiation	1–3 pumps daily	Cannot use to initiate puberty
E_2_: oral options		5 µg/kg/day		
17β-oestradiol (e.g.: Estrace, Cetura; Zumenon, Ormone, Estrofem mite, Estrofem)	0.5, 1, 2, 4 mg	Part of a pill daily^***^	1–4 mg/day	The cheapest option, brands vary by country
Oestradiol valerate (e.g. Climaval, Progynova)				
Ethinyloestradiol (EE_2_)		2 µg/day	10–20 µg/day	Not available in many countries
Premarin (CEE)	0.3, 0.625, 0.9, 1.25 mg	Part of pill daily	0.625–1.25 mg/day	Not available in many countries
Depot options				
Depot E_2_ (cypionate)	5 mg/mL	0.2 mg/mL	2 mg/mL	Not available in Europe
Adding Gestagen options		Not needed to initiate puberty		Add once bleeding occurs or after 2 years
Medroxyprogesterone acetate (e.g. Provera)	10 mg/tablet		Give with E_2_, or alone for 10 days/cycle	
Dydrogesterone (Duphaston)	10 mg/tablet		Give with E2, or alone for 10 days/cycle	
Micronised progesterone (e.g. Prometrium, Utrogestan, Progesterone Besins)	100 and 200 mg /tablet		Give with E2, or alone for 10 days/cycle	Utrogestan: before going to bed, lactose-free, indication: galactosemia
Progesterone (e.g. Luttagen, Luteina)				
Jaydess, Kyleena, Mirena	Intrauterine device		Give with E_2_	
Combined E_2_/Gestagen sequential patch		Do not use to initiate puberty		
Climara Pro	E_2_0.045 mg/levonorgestrel 0.015 mg/24 h		1 patch weekly	
Combipatch	E_2_0.045 mg/norethidrone 0.14 or 0.25 mg/24 h		1 patch weekly	
Evo-Sequi	E_2_50 µg/norethisterone acetate 170 µg/24 h		1 patch twice weekly	
Systen Sequi				
Combined E_2_/Gestagen sequential pills		Do not use to initiate puberty		
Trisequens	E_2_2 mg/norethisterone acetate 1 mg		1 pill/day	
Divina plus	Oestradiol valerate 2 mg/medroxyprogesterone acetate 10 mg		1 pill/day	
Femoston 1/10 or 2/10	Tablet 1–14: 1–2 mg E2; Tablet 15–28: 1–2 mgE2+ 10 mg dydrogesterone		1 tablet/day	
Femoston Continu	All tablets: 1 mg E2/5 mg dydrogesterone		1 tablet/day	
Oral contraceptive pills	Ethinyl estradiol and progestins	Do not use it to initiate puberty		
Loestrin (norethindrone)				Less progestational, less androgenic, low estrogenic
Lo-ovral (norgestrel)				More progestational, intermediate androgenic, low estrogenic
Orthotricylcen (norgestimate)				Less androgenic but progestational and more estrogenic

^*^Detailed comments are in the text; ^**^To avoid cutting (in daily practice, we cut the patches and inform our patients how to cut them; however, there is no manual in the product’s label); ^***^The preparation with the appropriate dose should be prepared by a pharmacist.

The optimal type, route of administration and dose of E2 used for female puberty initiation are not well established. In line, no clear advantage was found for any type of hormone treatment for puberty induction or to sustain puberty in girls. Patients using 17β-oestradiol had a higher chance to reach Tanner stage B3 during the study period (Fig. 2) than those with conjugated oestrogen, although the difference did not reach statistical significance.

Oestrogens can be used orally or transdermally for puberty induction. Their use is complicated by the lack of oestrogen formulations dedicated to younger patients. Thus, paediatricians have to deal with oestrogen formulations aimed for use by adult women (off-label). The possibility to split a transdermal patch, and thereby split the dose, facilitates mimicking the spontaneous increase in concentration, as well as the diurnal pattern of serum oestradiol in early puberty (135, 136).

Compared with oral E2, transdermal forms resulted in oestradiol, estrone and bioestrogen concentrations closer to normal in the high-dose transdermal group (137). The normalisation of gonadotropins was comparable after oral and transdermal oestrogen (138) but observed only after high-dose transdermal treatment (137). Data regarding the increase in the uterine size during oestrogen therapy are inconclusive, and only a few studies show that adult uterine volume can be achieved by using oral-conjugated oestrogens or oral contraceptives (139). Higher oral 17β-oestradiol dose for 5 years (2 mg vs 4 mg) in the years immediately after pubertal induction led to more girls with TS achieving a normal uterine size (140). See also Supplementary Appendix 3 Table 1.

The metabolic effects of transdermal and oral routes of oestrogen delivery are similar concerning multiple endpoints (bone mineralisation, body composition, BMI, lipids, glucose, insulin tolerance, protein turnover and lipolysis) (141, 142, 143), although it should be noted that firm evidence is lacking (see Supplementary Appendix 3 Table 1 for details).

Available evidence points towards a liver protective effect of E2 supplementation (144, 145, 146). Results regarding the influence of different routes of oestrogen therapy on IGF1 concentration are inconsistent (137, 142). Bone age advancement, one of the major concerns during oestrogen therapy, was less significant with transdermal oestrogen (147). Moreover, transdermal E2 compared with conjugated oral oestrogens resulted in faster bone accrual (spine) (104).

A randomised trial comparing transdermal and oral 17β-oestradiol and oral-conjugated oestrogen therapy in adolescents with ovarian failure did not show differences in fibrinogen and antithrombin activity, glucose and insulin, liver enzymes activity, lipids concentration, plasma renin, as well as IGFBP3 and IGF1 levels (105).

In TS, the long-term risk of breast cancer after long-term oral or transdermal oestrogen remains much lower than among control women (148).

**R 2.7**We recommend a follow-up frequency with a minimum of once every 3–6 months during pubertal induction or sex hormone replacement to sustain puberty in girls.

***Rationale***

When treatment is started, baseline values should be noted for parameters such as weight, height, Tanner stage, blood pressure, bone age and hormone measurements. The individual response to treatment and pubertal progress should be followed. Physical examination, including weight, height, blood pressure and Tanner stage should be assessed every 3–6 months to ensure the progress of puberty induction for each patient. Detailed history and discussion to monitor the compliance and resolve patient’s doubts regarding side effects of the therapy and its practical aspects (e.g. dosing, storage) (135) should take place during every visit. In case Müllerian structure is present, pelvic ultrasonography before the start of puberty induction, before adding progesterone or at the time of the first breakthrough bleeding is required. In the context of increasing options for fertility treatment, adequate uterine development and regular monitoring of its dimensions are recommended (119). The dosing should be adjusted accordingly (see below).

**R 2.8**We suggest titrating/adjusting the oestrogen therapy dose based on the appropriate progression of puberty during puberty induction or sex hormone replacement in girls. (+OOO)

***Rationale***

The main goal of all therapeutic protocols for puberty induction in hypogonadism should be to achieve an endocrine milieu similar to natural processes. For this purpose, a gradual increase over a period of 2–3 years until an adult dose of oestrogen is reached seems mandatory. Researchers used different schemes depending on their experiences, preferable administration route, patient’s age, medicine pharmacodynamics/pharmacokinetics and local availability. If the dose is increased too fast, it may have a negative influence on, for example, breast development or growth (149). However, no studies were found comparing titration or adjustment of hormonal therapy for puberty induction based on puberty progression.

For the initiation of oestrogen treatment with nocturnally administered E2 patches, the starting doses can be as low as 0.05–0.07 µg/kg, to mimic E2 levels during gonadarche. In older girls, when breast development is of high priority, the starting dose can be 0.08–0.12 µg/kg. Serum E2 levels of 17–23 pmol/L were found for doses of 0.05–0.07 µg/kg and E2 levels of 26–39 pmol/L on doses of 0.08–0.12 µg/kg (150). In turn, a 5-year study with oestradiol gel showed that the initial percutaneous dose of 0.1 mg ending at 1.5 mg leads to mean serum oestradiol concentrations increasing from 22.2 pmol/L at baseline to 162.2 pmol/L and mean FSH levels decreased from 77.4 IU/L at baseline to 19.2 IU/L after 5 years (151), which indicates that this dose is too small. Regular monitoring of oestradiol or FSH or LH during hormonal replacement therapy may be useful to guide treatment in addition to the full clinical picture, especially if mass-spectrometry is available for E2 measurements in the low ranges.

Most of the published experiences are from studies on patients with Turner syndrome (104, 126, 136, 151, 152, 153, 154). However, due to differences in puberty induction protocols, comparison between protocols is difficult. Nevertheless, the dynamic of breast development seems similar in most of the studies: stage B2 was reached during the first months and B4 during or after approximately 2–2.5 years (104, 152, 154, 155), a pace that is comparable to the pace of spontaneous puberty (2, 5, 6).

Low-dose oral oestradiol therapy given as a fixed dose (0.2 mg/day during the first year followed by 0.5 mg/day during the second year) is well-tolerated, not interfering with growth, and produces satisfactory pubertal development in patients with TS not inferior to individualised dose (5–15 μg/kg/day during 2 years) (153). A number of different oestrogen dose titration models for female puberty induction have been proposed (119, 156).

**R 2.9**We recommend that progesterone is added after puberty induction or during sex hormone replacement to sustain puberty in girls after breakthrough bleeding, after at least 2 years of treatment. (+OOO)

***Rationale***

If a uterus is present, progesterone must be added at some point because of the risk of endometrial cancer associated with long-term unopposed oestrogen (157). Progestins, synthetic progestagens, are most frequently used. Progesterone is the only bioidentical progestagen. Progestagens, similarly to oestrogens, can be used orally, vaginally, transdermally, intranasally or intramuscularly. There is a lack of valuable data concerning the optimal progesterone induction scheme; no studies were found comparing the addition of progesterone after or during puberty induction.

If optimal breast and uterine maturation has been achieved, it is assumed that progesterone should be added at the time of the first breakthrough bleeding or at least 2 years after oestrogen therapy initiation. In most protocols, a 10-day treatment given cyclically is preferred, as no evidence exists for the optimal duration (from min. 5 to max. 14 days). Oral, natural micronised progesterone (100–200 mg daily), oral dydrogesterone (10 mg daily) or medroxyprogesterone acetate (5–10 mg daily) or norethisterone (1 mg daily) and other preparations can be used (156, 158).

**R 2.10**We recommend to change pubertal induction treatment of oestrogen and progesterone to permanent adult sex hormone replacement therapy at the end of pubertal induction.

***Rationale***

The 2- to 3-year puberty induction is followed by the regimen of sex hormone therapy required in a young woman. Similar to puberty induction, transdermal or oral E2 should be the first choice; however, other, less recommended, options include EE2, depot E2 or equine-conjugated oestrogens (the last two are available and are used in the United States). Suggested adult dosing for the preparations is presented in Table 3 (119, 120). Decision regarding the optimal adult regimen is based on clinical features (effectiveness, tolerance), E2 levels and economic considerations. The presence of a uterus obliges to include progestogen in the regimen. However, there are no data clearly indicating the optimal route and regimen in female hypogonadism. Progestagens can be given cyclically or continuously, orally as a single or a component of contraceptive pills, transdermally combined with oestradiol and by intrauterine devices.

**R 2.11**Puberty induction in late-diagnosed patients must be individualised. A faster than normal increase in oestrogen doses can be considered in such cases.

***Rationale***

There are controversies concerning the optimal model of female puberty induction in patients with a delayed diagnosis or postponed initiation of oestrogen treatment. Late-onset puberty inductions need individualised approach to optimise the overall outcome with respect to patient’s wishes, height, secondary sex characteristics development and psychosocial endpoints.

The model of a faster increase in oestrogen dosing was presented in single studies regarding patients with Turner syndrome. In one such pilot study using a 1-year protocol with transdermal E2 therapy in 14-year-old girls (first 6 months 25 μg/day, thereafter 37.5 μg/day), a progressive increase in the puberty stage was observed; Tanner stage 3 or 4 was reached after 1 year in all and breakthrough bleeding in four of six girls (104). Another study with patients older than 14 years of age and a comparable simple protocol (12.5 μg/24 h for the first 2 months, thereafter 25.0 μg/24 h until breakthrough bleeding) suggested that easy-to-use fixed-dose regimen for late-onset puberty induction allowed for a satisfactory rate of pubertal stage progression and did not influence the growth potential (152).

Intentional delaying pubertal induction did not improve final height (112, 113). However, postponing pubertal induction in girls who are diagnosed particularly late and in whom short stature is a major concern should be discussed with the family (112).

## Puberty induction in boys

### Introduction

The initiation of puberty at an age comparable with peers is essential for normal physiologic development, including secondary sex characteristics, bone, muscle, and social, sexual and psychological development. In general, constitutional delay of pubertal development is more common among boys than girls, which may contribute to an even further delay of diagnosis and treatment with pubertal induction in boys with gonadal deficiency due to a DSD. In some cases, puberty may start but not progress properly, in which case a hormonal substitution is needed. A timely induction of puberty with testosterone in some form is appropriate. In case of pituitary deficiency with HH, alternative approaches are also possible (see R. 4).

#### In whom to consider puberty induction?

**R. 3.1**We recommend expert evaluation of puberty in any boy who has delayed puberty as defined by a puberty s.d. score <−2 or no signs of puberty at the age of 14 years or fails to show adequate progression in puberty.

***Rationale***

The start of puberty in boys is defined by testicular growth ≥4 mL. The timing of puberty is physiologic if the age at which it occurs is within 2 s.d. of a reference population (puberty nomograms exist for Caucasian boys (159)). Although puberty usually starts before the age of 14 years in boys, its timing is influenced by several factors including ethnicity, genetics and factors such as obesity and nutrition (160).

Expert evaluation of puberty in boys with suspected pubertal delay starts with a detailed history (161) and the initial evaluation aims to differentiate CDGP from other forms of hypogonadism that may be permanent or secondary to other systemic diseases (116, 162, 163). Clinically, the pubertal stage is measured by scoring Tanner stages and testis volume using a Prader orchidometer. Growth velocity, biochemical testing of gonadotropins and sex steroids and radiological evaluation of bone age by X-ray of the left hand give additional information. The family history on the timing of pubertal development is essential in the evaluation process. CDGP is the most common cause of delayed puberty in teenage boys (~63%), with a majority (50–75%) of them having at least one parent with a history of delayed puberty (117, 164). The diagnosis of CDGP can only be made after exclusion of conditions such as CHH or primary gonadal failure that leads to permanent hypogonadism, as well as chronic illnesses, or a tumour that may lead to a variable extent of delayed growth and puberty (115, 116). Depending on age, gonadal failure can be confirmed biochemically by hypergonadotropic hypogonadism. A history of cryptorchidism (especially bilateral), micro-penis, midline defects, hypo or anosmia, deafness or renal anomalies increases the likelihood of CHH. Furthermore, complications such as failed orchidopexies in boys with CHH can produce a complex biochemical picture that needs careful interpretation. CDGP can often be difficult to distinguish from CHH which may also be associated with a positive family history (115, 116). In addition, to a variable degree, CHH may be associated with other clinical features and an expert evaluation should explore this (115, 116). Clinically, CDGP is transient and confirmed by a spontaneous progression of puberty.

Although a karyotype or a chromosomal microarray is not routinely performed in boys with delayed puberty, this should be considered in those boys who have hypergonadotropic hypogonadism and do not have a predisposing condition such as a past history of testicular damage. Approximately 25% of boys with XY DSD have additional congenital malformations (165) and these boys are more likely to have copy number variations (166). Sex chromosome abnormalities, such as Klinefelter syndrome or 45,X/46,XY, may be associated with several distinct clinical features including behavioural problems, cardiovascular problems and increased sitting height percentage. Klinefelter is associated with tall stature and 45,X/46,XY with short stature (167). Thus, boys with delayed puberty and a past history of atypical genitalia, congenital malformations, hypergonadotropic hypogonadism or clinical features of sex chromosome disorders should also have a karyotype or a chromosomal microarray.

**R 3.2**All boys with a history of a condition that places them at risk of hypogonadism should undergo pubertal assessment from the age of 9 years.

***Rationale***

Several conditions that are associated with primary hypogonadism may present in early infancy with atypical genitalia. This includes sex chromosome disorders, disorders of gonadal development, disorders of androgen synthesis and lastly, disorders of androgen action. In these boys, a firm aetiological diagnosis of their DSD that is supported by endocrine and molecular genetic tests allows targeted follow-up that is appropriate for that specific diagnosis. In some conditions such as anorchia, in the absence of a specific aetiological diagnosis, the prospect of not undergoing spontaneous puberty is also unequivocally clear. The likelihood of developing hypogonadism is clearer for some conditions than for others, but ensuring that all these boys are followed up as per current recommendations in a standardised manner will ensure improved knowledge in the future (168). Klinefelter syndrome is the most common cause of congenital male hypogonadism, and although these boys typically enter puberty at a normal age, they often have signs of gonadal dysfunction including gynaecomastia, small, firm testes, elevated gonadotropins and relatively low testosterone levels (169, 170). These boys may also, in rare cases, have a past history of atypical genitalia and an ongoing history of learning difficulties or difficulties in social adjustment.

In 45,X/46,XY sex chromosome mosaicism, spontaneous puberty has been reported to occur in 80% but may be less likely in those who present in the neonatal period with atypical genitalia. Over 50% of this ‘early presentation’ group (diagnosed due to genital anomalies and/or short stature) required testosterone supplementation in puberty compared to 15% in the late presentation group (diagnosed in adulthood due to infertility work up) (25). In partial gonadal dysgenesis, a disorder of gonadal development, absent pubertal development has been reported in 10–15% of adolescents (22). In the rest who have spontaneous initiation of puberty, the gonadal function may not be sufficient to sustain pubertal development, and most of the adolescents with partial gonadal dysgenesis demonstrated elevated gonadotropins with only about half having a serum testosterone in the normal range after puberty (22).

In patients with steroidogenic factor 1 ( SF1) deficiency, Leydig cell function can be effective in puberty, even in patients who were severely under-virilised at birth (171). Testicular function may deteriorate over time. Boys with 46,XX testicular DSD who present with atypical genitalia often have primary hypogonadism. On the other hand, men who present to an assisted conception service and are discovered to have 46,XX karyotype rarely have a history of atypical genitalia in infancy but are infertile, due to the absence of the azoospermia factor genes on the Y chromosome (33, 172). Testicular function in boys with ovotesticular DSD can be quite variable and depend on the androgen-producing capacity of the testicular component of the gonads (34).

In conditions that are due to a genetic disorder of androgen synthesis, boys can undergo a variable range of spontaneous virilisation in puberty but may require topical or systemic androgen supplementation. In many patients with DSD, especially partial androgen insensitivity syndrome or Klinefelter syndrome, the affected boy may display additional features such as gynaecomastia, disordered growth or even precocious puberty.

The monitoring of pubertal development includes both clinical measurements and laboratory assessments. Clinically, assessment of growth, Tanner stage and testicular volume with a Prader orchidometer are useful to detect pubertal onset and laboratory assessment includes the assessment of LH, FSH, total testosterone measurements, inhibin B and AMH. In some forms of primary testicular failure, a differential effect of the Leydig cell and Sertoli cell compartments within the testis may make the assessment of testicular volume misleading. In cases of selective Sertoli cell failure and/or lack of germ cells, testicular volume usually remains small although testosterone production may be active. Boys with partial hypogonadism may also show signs of precocious puberty (173, 174).

As a greater number of infants with XY DSD are being raised as boys nowadays (175), it is likely that there will be a greater number of boys with a wide range of partial forms of hypogonadism who approach pubertal age and will need regular support. Thus, to see these boys at around 9 years allows the DSD team to gauge the need for clinical and psychological support as the patient proceeds through adolescence. Starting pubertal monitoring at a relatively early age provides the physician time to specify and explain the diagnosis and to make shared decisions with patient and parents on therapeutic options.

**R. 3.3**We recommend that all boys with permanent hypogonadism should undergo pubertal induction.

***Rationale***

Androgens are required to induce secondary sexual characteristics, achieve optimal adult male body composition including bone mineral content and muscle mass and promote physical and social well-being. Testosterone plays an important role in socio-emotional and cognitive development (176, 177). In boys with pre-existing behavioural or intellectual disabilities, pubertal induction should be performed carefully and in close collaboration with a mental health specialist. Parents and caretakers of such boys should be informed about the effects of testosterone on impulsivity. In addition, pubertal induction should be accompanied by sexual education and by the provision of resources for sex education elaborated for individuals with intellectual disability. Studies show that this specific patient group remains too often insufficiently educated and prepared about sexual and emotional life situations (178).

In boys with HH, there is a wide range of pubertal induction regimens, including the use of gonadotropins that are available, see section on ‘hypogonadotropic hypogonadism’. There is no clear evidence that one regimen is superior to another and there is no clear evidence that supports the preferential use of any of these regimens instead of testosterone replacement for the purpose of achieving virilisation in boys with HH. The preparations used will depend on the preferences of the patient, ease of use as well as local availability and if fertility is considered.

**R. 3.4**In boys who are known to have a diagnosis with a high risk of hypogonadism, we recommend that pubertal induction can be started by the age of 12 years if there are no signs of pubertal development. (+OOO)

***Rationale***

The aim of pubertal induction in boys with hypogonadism should be to mimic normal physiology. Attention needs to be paid to the age-appropriate development of secondary sexual characteristics, growth acceleration, normal body proportions achievement, changes in body composition, avoidance of psychosocial consequences of delayed growth and puberty. Late-onset of puberty has been associated with a higher risk of anxiety and depression (179) and increased cardiometabolic risk (180). There are also ethnic and familial variations in the onset and tempo of puberty, which may need to be considered when deciding to start pubertal induction in an individual patient. For instance, pubertal onset as determined by age at testicular volume of at least 4 mL varies from 10.6 years in the Chinese population to 11.4–11.7 years in Hong Kong, the Netherlands, Greece and Denmark (181).

Surveys performed in the context of managing CDGP have shown that in paediatric endocrinology, pubertal induction in boys is usually started after the chronological age of 14 years (169). However, no studies were found comparing different age groups for the start of puberty induction in boys with (high risk of) hypogonadism (see section on Systematic literature review). Factors that may influence the timing of induction may include the bone age and the sociocultural perspective (182). In boys with conditions that predispose to permanent hypogonadism, such as bilateral anorchia or known CHH, surveys of practice suggest that pubertal induction may often be initiated at an age younger than 12 years and even as young as 10 years (164, 183). These studies also show that in several boys with DSD conditions associated with hypogonadism, testosterone therapy may be initiated at a later age possibly due to a partial hypogonadism in these boys.

**R. 3.5**We recommend that pubertal induction in boys with hypogonadism should be performed with testosterone (++OO)

***Rationale***

There is a wide range of testosterone preparations that are available for use in boys with hypogonadism (see Table 4). Recent studies show that in boys with early onset hypogonadism, i.m. forms of testosterone remain the commonest form of preparations (164). Studies are scarce that compare different forms of testosterone (see section 4.1), but there is no clear evidence that one form of testosterone is superior to another (184, 185). One study comparing testosterone vs no treatment to induce puberty found increased BMD in patients using testosterone compared to the control group (100). One study comparing testosterone vs hCG for puberty induction showed inconclusive results regarding Tanner stage progression, smaller testicular volume and similar height after use of testosterone compared to hCG (101).

**Table 4:** Testosterone preparations (refer: (189, 190)).

	Starting dose for pubertal induction	Adult dose	Advantages	Disadvantages
Intramuscular				
Testosterone enanthate, cypionate or mixture of T esters	25–50 mg monthly. Increase of 50 mg every 6–12 months	150–250 mg every 2–4 weeks	Good adherence; most data and clinical experience to support use in adolescents	Not physiological; painful
Testosterone undecanoate, i.m. injection	No data available	750–1000 mg every 10–14 weeks	Stable serum T concentrations; less frequent injections	Painful injections; expensive; lack of data in adolescents
Transdermal				
Testosterone gel	2%: 0.5 g 10 mgT/day	2%: 2–4 g 40–80 mgT/day	Mimics normal physiology; pain free	Potential transfer to another individual
Testosterone patch (Scrotal)	No data available	4–6 mg/day	Mimics normal physiology	Skin irritation; patch is too large for prepubertal boys; lack of data in adolescents
Testosterone patch (non-scrotal)	2.5 mg × 12–24 h/day or 5 mg × 8 h/day	2.5–5 mg/day	Mimics normal physiology	Skin irritation; lack of data in adolescents
Oral				
Testosterone undecanoate (Restandol)	40 mg alternate day or daily	40–80 mg 2–3 times daily	Oral; pain free	Multiple doses needed per day; variable absorption
Testosterone undecanoate (Jatenzo)	No data available	158–396 mg twice daily	Oral; pain free	Multiple dose; GI side effects; concerns rehypertension
Buccal testosterone	No data available	30 mg twice daily	Mimics normal physiology	Altered taste; gum irritation
Subcutaneous				
Testosterone cypionate	25 mg subcut every alternate week	50–70 mg subcut every week	Less painful than i.m.; can be administered at home	Lack of data in hypogonadal boys
Testosterone pellets	8–10 mg/kg once	3–4 pellets (75 mg each) every 4–6 months	Adherence	Risk of extrusion of pellet, fibrosis, infection Cost
Intranasal				
Intranasal testosterone	No data available	11 mg tid	Non-invasive; easy to administer; no transference	Altered taste

In healthy boys, testosterone measured by LC-MS/MS starts to increase a year before the appearance of pubic hair growth, at 11.5 years on average. When measured by RIA, plasma testosterone starts to increase at a testicular volume between 3 and 6 mL with clear evidence of diurnal variation at a median age of 12.5 years (186, 187). While mimicking the gradual physiological rise in testosterone remains the objective of pubertal induction, it is unlikely that the wide range of pharmacological preparations that are currently available possess the pharmacodynamic and kinetic properties to achieve completely normal pubertal development, especially in the early stage of puberty. As an example, measured plasma testosterone after 25 mg testosterone reached an adult range for 1 day and for 2 days after 50 mg testosterone enanthate (188).

For suggested testosterone formulations and doses, see Table 4. The addition of DHEAS in boys with hypogonadism is not recommended. Although the benefits of mimicking normal physiology or, indeed, the adverse effects of not mimicking normal physiology have not been studied thoroughly, it is clear from boys with precocious puberty that quick progress is associated with adverse behaviour as well as restricted growth prognosis. Thus, the dose of testosterone should be increased gradually to mimic normal growth and pubertal development. It is common to begin treatment with low-dose testosterone (i.e. 50 mg testosterone enanthate every month, 10 mg transdermal testosterone every other day or 40 mg oral testosterone undecanoate daily) and then gradually increase to the adult dose. The speed of this gradual increase will also need to be individualised based on the age as well as the mental maturity of the boy.

**R. 3.6**We recommend that all boys who receive pubertal induction therapy should have a structured endocrine assessment at baseline and at follow-up.

***Rationale***

Regular clinical follow-up is generally performed every 3–6 months to assess the effectiveness of testosterone therapy by assessing pubertal and skeletal maturation and height velocity. In the guidelines for adults on testosterone replacement therapy (TRT), monitoring is recommended and standardised (191, 192). There is clear evidence that systematic monitoring in adolescents on testosterone is performed to a variable extent (164) and it is possible that this variation is due to the wide range of conditions that necessitate TRT in adolescents. Recently, monitoring schemes designed to assess the effectiveness as well as safety of TRT have been proposed (183). With greater knowledge of a wider range of effects of testosterone and the advent of several newer forms of testosterone replacement, the need for careful monitoring is becoming greater. A structured assessment can also be used to screen for adverse effects as well as titrate replacement. It should include the assessment of adverse signs such as local reactions, gynaecomastia, priapism, increased haematocrit, deranged liver function and inappropriate behavioural changes. Table 4 highlights the advantages and disadvantages of specific preparations and further details can also be found in recent publications (183, 189, 190). While there is no evidence that dose titration against a marker such as a haematocrit is necessary for pubertal induction, the regular assessment of haematocrit would be advisable, especially as the adolescent reaches a steady adult replacement dose (183). The care pathway can also ensure age-appropriate explanations and discussions about both endocrine and reproductive testicular functions with the appropriate experts in the multidisciplinary team. With increasing maturity, these discussions may cover issues regarding sexual function and fertility including biological fathering and other modes of fathering. Physiological variations in steroid metabolism as well as androgen sensitivity when combined with the wide range of preparations that are available increase the likelihood of inter-individual differences in how boys will respond to replacement therapy and there is a need for clinically relevant markers of androgen action.

**R. 3.7**We suggest adjusting the puberty induction treatment or changing the route of administration in case of relevant adverse effects/complications.

***Rationale***

The occurrence of relevant adverse effects is rare with testosterone (193). For injectable forms of TRT, priapism has rarely been reported. Testosterone undecanoate, the long-acting preparation, should not be used for induction of puberty or progression through the early stages of puberty, as it is likely to advance bone age too rapidly with a potential for truncating final height.

When ingested orally, testosterone is absorbed well from the gut but is effectively metabolised and inactivated in the liver before it reaches the target organs (‘first pass-effect’). Testosterone induces liver enzymes that are responsible for its own metabolism.

Testosterone gel provides a possibility for more physiologic serum testosterone levels and it can closely mimic natural diurnal variation in testosterone concentrations. It has only minor systemic side effects. However, published data on transdermal testosterone in pubertal induction are scarce. In many countries, testosterone formulations are not licenced for the induction of puberty.

Transdermal gels are available in different formulations: 1% testosterone strength and the metered-dose gel formulation of 2% or 2.5% testosterone strength. Topical testosterone has been repeatedly reported to cause undesirable exogenous testosterone exposure through passive transfer to members of the patients’ household (194). Due to the alcoholic enhancer used and the occlusive nature of the systems, the application is associated with skin irritation in up to 60% of the subjects.

**R 3.8**We suggest monitoring potential gynaecomastia in boys to timely discuss treatment options.

***Rationale***

Testosterone therapy can lead to gynaecomastia through its aromatisation to oestradiol; hence, regular monitoring of this potential side effect is requested. Gynaecomastia occurs in up to a third of patients on gonadotropins (195) or testosterone (196) and usually (although not invariably) occurs during supraphysiological replacement doses. The risk and severity of gynaecomastia can be reversed by adjusting the dosage. It should be remembered that 50% of all adolescents develop gynaecomastia which typically presents in early puberty and lasts for 6–12 months.

Similarly to physiological puberty, the most likely cause of gynaecomastia during TRT is a relative imbalance between oestrogen and androgen levels in the serum or at the tissue level (197). During the early proliferative phase, manifested clinically as breast pain and tenderness, medical therapy may be attempted (198). Medical treatment of gynaecomastia aims to correct the oestrogen–androgen imbalance by three possible pathways: (i) blocking the effects of oestrogens on the breast (e.g. clomiphene, tamoxifen, raloxifene), (ii) administering androgens (e.g. danazol or DHT) and (iii) inhibiting oestrogen production (e.g. anastrozole, testolactone). Almost all data dealing with these treatments are based on uncontrolled studies, and in addition, the evaluation of their efficacy is further complicated by the fact that gynaecomastia may resolve spontaneously (199, 200). It must be noted that none of these therapies are approved for the treatment of gynaecomastia. It seems to be reasonable to try a 3 months trial in selected cases of recent-onset gynaecomastia (201) because it offers rapid relief from pain, regardless of the magnitude of the response. If the gynaecomastia has been present for >1 year, it is unlikely to regress substantially, either spontaneously or with medical therapy, because fibrotic tissue is usually present. In such circumstances, surgical s.c. mastectomy, ultrasound-assisted liposuction and suction-assisted lipectomy are the best options for cosmetic improvement of Tanner stage III and above (202, 203).

**R 3.9**Treatment in patients with a late diagnosis, without pubertal development or for whom the pubertal development has come to a halt, should be individualised.

***Rationale***

Patients who come to diagnosis at a later age, towards the late teenage years or as young adults, may have no pubertal development or a spontaneous start of puberty but a slow or no pubertal progress. This is often the case in young men with Klinefelter syndrome and can be the case in individuals with 45,X/46,XY, gonadal dysgenesis or androgen insensitivity.

Adult endocrinologists often see patients with CHH in late adolescence or early adulthood with the main complaint of lack of pubertal development (204). In such cases, the therapeutic approach is often more incisive than for younger patients, involving higher initial testosterone doses than those used by paediatric endocrinologists (200–250mg testosterone enanthate monthly, then every 2–3 weeks) to induce more rapid virilisation (205).

### Pubertal induction in patients with hypogonadotropic hypogonadism

#### Introduction

HH is caused by congenital or acquired defects of the hypothalamic and/or pituitary compartment of the HPG axis. CHH forms can be isolated, part of an overlapping syndrome (such as CHARGE) or part of combined pituitary hormone deficits. Kallmann syndrome is associated with hypo- or anosmia. All these forms have strong genetic backgrounds (206). Acquired forms of HH, mainly associated with other pituitary hormone deficits, are usually the results of neoplastic, metabolic, immune, infectious, infiltrative, inflammatory or iatrogenic causes affecting the hypothalamic-pituitary region. HH can also be related to functional causes, which are usually characterised by a reversible negative effect on the HPG axis activation/functionality. These include chronic and/or inflammatory systemic illness, malnutrition (e.g. anorexia nervosa), extreme exercise or stressful conditions and opioid use.

Pubertal induction has to be considered in all cases with permanent HH after the diagnostic procedure has been carefully followed. The goals of the therapy for pubertal induction in male and female HH are not different from the ones reported for primary hypogonadism, although in this specific cohort of patients, the possibility of future fertility can be considered and, in males, specific treatment regimens can allow for the maturation of testes (207, 208). Briefly, the main goals will be the attainment of optimal masculinisation/feminisation and secondary sexual characteristics; the achievement of pubertal growth and an optimal target height; the prevention of osteoporosis by accruing normal bone mass and mineralisation; to allow for a normal psychosocial development; and in certain cases, to gain fertility (20, 207, 208, 209).

#### Timing of puberty induction

In principle, the timing for pubertal induction in patients with a diagnosis of permanent HH does not differ from what has been recommended for boys and girls with hypogonadism. However, it should be pointed out that the main differential diagnosis of CHH is CDGP. It is a more common situation and also more frequent in boys compared to girls. This differential diagnosis is one of the greatest challenges for the clinician evaluating a pubertal delay (116). Unfortunately, to date, we do not have specific indicators that allow for a clear distinction between these two clinical conditions although a history of micropenis, bilateral cryptorchism, midline defect, hypo/anosmia, deafness or renal anomalies should raise the suspicion of CHH. For this reason, in patients with pubertal delay and uncertainty regarding the diagnosis of CHH or CDGP, it may sometime be necessary to delay the initiation of induction even beyond 14 years. Nonetheless, the decision must always be made according to the clinical situation in each individual case and in concern with the patient him/herself and his/her parents (20, 205, 209).

#### Treatment approach and monitoring

Recommendations regarding treatment approach and monitoring for puberty induction in girls (R 2.1–2.9) apply equally to those with HH. Pubertal induction in girls with HH using gonadotropins has not been described, although gonadotropin–ovulation–induction is a standard fertility treatment in adult life.

**R 4.1**We suggest discussing two treatment options for pubertal induction in boys with secondary hypogonadism: testosterone or gonadotropins. See Table 5 for the so far reported different treatment options using gonadotrophins. (+OOO)

**Table 5:** Treatment with gonadotropins for puberty induction or induction of spermatogenesis.

Age (years)	*n*	First-phase therapy dose frequency	Treatment length (months)	Second phase or unique phase therapy dose frequency		Treatment length (months)	Reference
				rFSH or hMG	hCG or GnRH		
18–34.5	16	hCG (*n* = 12): 1.5–10.000 UI/w	1–14	hMG: 225–750 UI; ?/week	hCG: 3–15 000 IU; ?/week	18–28	(221)
12.8–13.2	3			rFSH: 1.5 U/kg; 3/week	–	12	(219)
13–15.2	37	hCG: 1500 UI 2/w s.c.	6	hMG: 75 UI; 2/week	hCG: 1500 UI 2/week s.c.	24	(222)
13.7–21.1	14			rFSH: 75–100 UI alternative day/week	hCG: 1000–1500 UI; alternative day/week	na	(217)
18–31	8	rFSH: 150 UI/day s.c.	1	rFSH: 150 UI/day s.c.; *n* = 7	hCG: 1500 IU i.m.; 2/week	2	(223)
10.4–17.7	14	rFSH: 1.5 U/kg (180–450); 3/week s.c.	2–32; *n* = 9 >12	rFSH: (no dose specified)	hCG: 500–4000 s.c./week; 1–3/week (?)		(215)
14.5–31	19	*n*= 8: testostosterone	6–36	rFSH; 150–300 UI 3/week^*^	hCG: 500–1500 UI; 2/week (increments 6 months)	9	(218)
Adults	18	rFSH: 75–150 IU/day	4		GnRH pulsatile	24	(214)
18–45	67	Groups A & B: hCG 2000 UI; 2/week	6	Group A: uFSH 75 UI; 3/week Group B: uFSH 75 UI; 3/week 3 months alternative	Groups A & B: hCG 2000 UI; 2/week	18	(224)
Groups	60						(225)
A: mean 15.5	34	hCG: 250–500 UI 2/week s.c.	250–500 UI increase every 6 months	rFSH: 75–150 UI 3/week (max 150 UI)	hCG: max 2500 UI 3/week	24 ± 7	
B: mean 18.8	26	Testosterone (previous to study)	18–68.4; mean 30	rFSH: 75–150 UI 3/week (max 150 UI)	Start 1500 IU s.c. 2/week → max 2500 UI 3/week	22 ± 6	
14.8–15.1	2	rFSH: 66.7–112.5 UI; 3/week	6–7	rFSH: 66.7 UI; 3/week	hCG: 500-1500 UI; 3/week	33–34	(220)
14–23	19	FSH (13/19 patients): 75 IU e/week	4	FSH: 75 IU 3/week	hCG: 250–2000; 2/week (increments 6 months)	24	(216)

hCG, human chorionic gonadotropin; hMG, human menopausal gonadotropin; rFSH, recombinant follicle stimulating hormone; GnRH, gonadotropin releasing hormone.

***Rationale***

Pubertal induction in a boy can be carried out through two different possible approaches: the use of testosterone analogues similar to the treatment of primary hypogonadism or the use of exogenous gonadotropins. Preparations, doses and aim of the two possible treatments are indicated in R 3.1–3.5 and Tables 4 and 5. Monitoring of patients with HH undergoing gonadotropin treatment does not differ from the recommendation for pubertal induction with testosterone (see R 3.5–R 3.8).

To date only a few randomised studies have been published comparing these different treatment strategies and the patients recruited in these studies are often quite heterogeneous. It is therefore difficult to conclude which treatment strategy is more effective or preferred (210). A systematic review provided no firm evidence for an optimal induction approach (see section on Systematic literature review) (100, 101, 102, 103). The two options, testosterone or gonadotropins (Table 5), should be presented and discussed with the patient and his parents, also considering the availability of the different treatment options in the patient’s country of residence.

The most common treatment for pubertal induction in males with HH involves, as for primary hypogonadism, the use of i.m. injection of testosterone esters (enanthate, propionate or cypionate) (20, 205, 209, 210, 211). Although effective in inducing pubertal signs and symptoms, testosterone formulations neither stimulate testicular growth nor will they induce spermatogenesis. It is still unclear whether testosterone therapy for pubertal induction hinders future testis development or likelihood of spermatogenesis if gonadotropins are used in adulthood (212, 213).

Alternatively, a potential testicular development and fertility in males with HH can be achieved with puberty induction through the use of gonadotropins (212, 214, 215, 216). This might offer adolescent patients a significant psychological reassurance and may also enhance their self-confidence (20, 205). On the other hand, although this has to be verified and demonstrated, it might also allow for the early identification of potential ‘poor responders’ (i.e. patients with severe oligozoospermia and a total sperm count <5 millions/mL) and thus give them the opportunity to consider a preventive and prophylactic sperm cryopreservation for future use in case of a worse spermatic response in adulthood. Different treatment protocols with gonadotropins can be used to induce puberty in adolescent males with HH: exogenous hCG alone or in combination with recombinant FSH (214, 215, 216, 217, 218). However, consistent evidence suggests that the combination therapy with recombinant FSH (rFSH)/hCG is significantly more effective than hCG alone both for inducing spermatogenesis and increasing testicular volume. Furthermore, some evidence suggests that a pre-treatment with rFSH followed by the combination with hCG or GnRH is even more effective in optimising the Sertoli cell maturation and is able to induce spermatogenesis even in extremely small testes, and cryopreservation of sperm subsequently allows for enhancing the future fertility of these patients (214, 215, 219, 220). Indeed, this approach mimics the physiological elevation of FSH during spontaneous pubertal development.

Treatment with gonadotropins, whatever the protocol used, requires multiple weekly injections, although s.c. injections can be given. This aspect should be discussed with the patient and parents before the final decision.

**R 4.2**We suggest considering switching gonadotropins to testosterone in boys with HH after pubertal induction.

***Rationale***

If pubertal induction is made by the administration of rFSH and hCG in order to mimic physiology, we recommend switching to testosterone treatment in boys with HH after the completion of pubertal induction. Possible spermatogenesis should be considered. Although there is not enough evidence to recommend one treatment before the other, there are theoretical arguments based on the limited knowledge available on testis and Sertoli cell maturation with regard to fertility. During early puberty, the proliferation of immature Sertoli cells occurs under the stimulus of FSH. Hence, treatment with FSH stimulates the proliferation of immature Sertoli cells and spermatogonia (214, 219, 226). On the other hand, testosterone induces the differentiation of Sertoli cells via androgen receptor which begins to be expressed after the first 5 years of life (211, 227). The combination of rFSH and hCG therapy for 6–24 months results in testicular growth in almost all and spermatogenesis in 80–95% of patients without undescended testes (216, 217, 228, 229, 230). The use of hCG without FSH priming could, therefore, in theory, cause premature differentiation of the pool of Sertoli and germ cells, thereby diminishing the chance of proliferation leading to lower fertility potential in adulthood. The shift from FSH/hCG therapy to testosterone should occur after sperm analysis, and in the case of sufficient sperm in the ejaculate, sperm cryopreservation (banking) should be considered, particularly in severe oligospermia. The reason behind this is that even if repeated gonadotropin treatment has been reported to induce quicker initiation of spermatogenesis (231, 232), it is not certain. In cases with azoospermia, which then appear as ‘poor responders’ to gonadotropin stimulation, testicular sperm extraction (TESE) procedure might be considered, however, with uncertain outcome, before switching to TRT, especially in patients who are old and mature enough for this.

Furthermore, since ‘reversal’ of CHH has been described (144) in 5–20% of patients with CHH, a brief therapy withdrawal (i.e. 4 months) after the conclusion of pubertal induction with gonadotropins and before the start of TRT might be considered in order to verify a spontaneous recovery of the HPG axis.

### Puberty induction in Klinefelter

#### Introduction

Klinefelter syndrome is the most common cause of congenital male hypogonadism. However, boys with this syndrome typically enter puberty at a normal age. With advancing adolescent age, the gonadal dysfunction becomes more marked and may be associated with a failure to progress in puberty (169). The diagnosis of Klinefelter syndrome affecting 1:600 men is often overlooked (233, 234) with less than 10% diagnosed before puberty, probably due to the variable and sometimes subtle phenotype, especially in the mosaic forms. During or after puberty, boys with Klinefelter syndrome typically present with gynaecomastia and they have elevated FSH and low testosterone levels, although testosterone can also be at the lower end of the normal range with elevated LH (167, 169). Patients have an increased incidence of cryptorchidism. In puberty, the testes grow initially and then regress, and in adulthood, testes are small and firm. There is a wide spectrum of the phenotype, many are never even diagnosed while some can have psychosocial difficulties and learning disabilities. The diagnosis is confirmed by karyotyping.

We place a high value on the recommendation to treat adult patients with Klinefelter syndrome and low testosterone levels as well as accompanying symptoms of androgen deficiency with an adequately dosed testosterone replacement (Table 4).

#### Timing of puberty induction

The general recommendations as formulated under R 3.1 and 3.3 (in whom to consider pubertal induction) apply to Klinefelter syndrome, although the most common situation is a normal start of puberty followed by a slow progression of puberty with small testes and development of gynaecomastia.

#### Treatment approach and monitoring

With regard to the timing of puberty, an individual prediction of pubertal onset for patients with Klinefelter syndrome can be estimated from the parents’ pubertal history. Whether early testosterone replacement, when LH starts to rise above normal levels (>+2 s.d.), should be started is still not clear. Some advocate for early treatment as soon as LH starts to rise in order to prevent the adverse body composition, osteopenia/osteoporosis, impaired physical and psychological development, educational achievements and social integration which are commonly observed in the untreated newly diagnosed young adult (170, 235, 236, 237, 238). However, others think that testosterone supplementation should be postponed until testosterone levels fall below the normal range (<−2 s.d.) (239). Some in the expert group advocated for aiming at securing sperm for cryopreservation before starting testosterone supplementation, while others, in the absence of scientific evidence for such an approach, advocated for commencing testosterone supplementation as soon as LH starts to rise.

The recommendations regarding treatment and monitoring R 3.5–3.9 apply equally to Klinefelter.

**R. 5.3**We suggest performing sperm analysis if physically and mentally possible before the start of testosterone treatment in boys with Klinefelter Syndrome and spontaneous start of puberty.

***Rationale***

Randomised–controlled trials are needed to elucidate the influence of testosterone replacement therapy on pubertal development and spermatogenesis in patients with Klinefelter Syndrome. There are also no randomised–controlled trials evaluating the possible deleterious impact of testosterone treatment on successful sperm retrieval or its possible effects on reproductive outcomes in men with Klinefelter Syndrome. Testosterone preparations may theoretically suppress any remaining spermatogenesis if the endogenous LH drive is reduced; however, this has not been shown in clinical trials. Some advocate for assessing fertility status before the start of testosterone supplementation, while others do not. Therefore, individual decisions should be made until more evidence is gathered. In those patients who have no spermatozoa in their ejaculate, fertility preservation can be attempted through TESE. According to a recent literature review, spermatozoa can be found by TESE in up to half of the patients with Klinefelter syndrome aged 16–30 years old, of whom many had been treated with testosterone (240), although real-life studies report much lower values (241). Therefore, a TESE procedure for fertility preservation should not be performed in Klinefelter syndrome patients younger than 16 years and it can be postponed at least until the age of 30 since no significant differences were observed in TESE outcome in this age interval.

The use of hCG or anti-oestrogens (aromatase inhibitors or selective oestrogen receptor modulators) on compassionate care grounds may have a positive effect on gonadotropin secretion even in the hypergonadotropic state as seen in Klinefelter syndrome (242). Theoretically, this may result in higher levels of intra-testicular as well as circulating testosterone and maintained (or even augmented) spermatogenesis. However, no controlled trials are available and evidence is poor (243). Hence, such therapies cannot be recommended in general.

No data have been published on the possible positive or negative effects of testosterone treatment in boys with Klinefelter syndrome and compensated hypergonadotropic hypogonadism. The guideline group acknowledges that different strategies are employed. Some suggest that in the absence of clinical signs and symptoms of hypogonadism, these boys should not be treated with testosterone (239). In some earlier studies, no benefit of TRT was observed in preventing obesity/overweight (238), and no significant differences in bone structure or bone biomarkers were reported in patients with Klinefelter with and without testosterone therapy (244). However, a recent study advocates testosterone treatment in cases with elevated LH in order to potentially positively affect the muscle mass, the BMD and neuropsychological functioning (170). In young adults with Klinefelter syndrome, a recent placebo-controlled, randomised trial has shown that 6 months of testosterone treatment led to decreased total body and abdominal fat mass, as well as expected increases in haemoglobin and IGF-I (245). In adults, individualised transdermal and injection testosterone therapy resulted in comparable treatment efficacy when evaluating androgen-responsive parameters (246).

### Androgen insensitivity syndrome (PAIS and CAIS)

Androgen insensitivity syndrome (AIS) is a rare inherited condition caused by X-linked pathogenic mutations in the androgen receptor (*AR*) gene (38, 247). There are now more than 1000 mutations reported (248), and the genotype–phenotype correlation is highly variable. The estimated prevalence is 1–5 per 100 000 births (249, 250). The phenotype depends on the residual AR function. According to the residual sensitivity to testosterone, the external genitalia will be variably virilised (251, 252, 253). In general, three subgroups of AIS are defined.

A complete absence of androgen receptor action leads to the clinical picture of CAIS. In these girls, the external genitalia are typically female. They have testes, which secrete testosterone with no (or markedly diminished) effect on the androgen receptor and Sertoli cells that produce AMH resulting in the regression of Mullerian structures prenatally. Thus, the uterus, fallopian tubes and upper part of the vagina are absent. Furthermore, Wolffian structures do not develop due to the insensitivity to testosterone. The testes are typically located within the abdomen, inguinal canal or in the labia majora (250). Girls and women present during puberty with absence of menarche but with normal breast development due to the presence of androgens which are aromatised to oestrogens. Some girls (up to 50%) present with a bilateral inguinal hernia during infancy/childhood (254, 255, 256). Women with CAIS do not develop pubic and axillary hair. A blind-ending vagina is observed in almost all patients (from 2.5 to 8 cm long). Women with CAIS have a female gender identity and are female in their gender role behaviour (66, 68).

Reduced AR residual activity results in PAIS with variable virilisation of the external genitalia during fetal life. Individuals generally present with ambiguous genitalia at birth. Earlier studies described a grading scheme (grade 1–5) for the description of virilisation (249). The residual activity of the AR also leads to a variable degree of virilisation of the external genitalia and the presence of gynaecomastia at the onset of puberty. Unfortunately, the phenotype–genotype correlation is poor (257). However, it has been shown that the degree of virilisation at birth estimated by the external masculinisation score (EMS) is a good predictor of the degree of virilisation at puberty (175, 258, 259). In only 22% of the cases with a clinical diagnosis of PAIS, a pathogenic variant in the AR is found, making it a more complex and a highly variable clinical and diagnostic challenge (258, 260).

The mildest form of androgen insensitivity called mild androgen insensitivity syndrome is typically diagnosed in men with infertility without atypical genitalia (261, 262, 263). A detailed description of this form is outside the scope of this guideline.

#### Gonadectomy in patients with complete androgen insensitivity syndrome

Patients with CAIS and intraabdominal testes might be at increased risk of developing gonadal germ cell cancers (GGCC), mainly seminomas, but this risk seems to be low before/during puberty (264, 265, 266). Historically, nearly all patients with CAIS underwent gonadectomy before puberty because of this risk. Delaying gonadectomy allows for spontaneous pubertal development which is thought to be more satisfactory to the individual and also allows the patient to be fully involved in the shared decision-making to remove their gonads or not. There is an ongoing debate about the need to perform gonadectomy after puberty since the risk of invasive GGCC development is still uncertain. In a recent publication by Tack et al., an algorithm was presented, in which they suggest performing gonadectomy after puberty in individuals with CAIS and only after careful counselling. Yearly follow-up if the gonads are retained was advised including self-examination and imaging (ultrasound/MRI) (265). However, self-examination is only possible when the gonads are located in the inguinal region.

Women with CAIS and intact gonads have an increased risk of developing low BMD due to bone insensitivity to testosterone and relative oestrogen deficiency. After gonadectomy, the risk of developing low BMD further increases. Treatment with sex steroids aims to prevent osteoporosis and other metabolic complications (267, 268, 269, 270, 271, 272, 273). Effects on cardiovascular health, quality of life, sexual health and mortality are less well studied (274, 275).

### Pubertal induction in CAIS

#### In whom to consider puberty induction?

**R 6.1**We recommend allowing girls with CAIS and retain gonads to go through spontaneous puberty.

***Rationale***

Puberty treatment is not indicated in girls with retained testes. In subjects with CAIS, androgen-responsive tissues including the pituitary gland are insensitive to testosterone, resulting in persistently elevated LH concentrations. Testosterone levels are within or above the normal male range in patients with retained testes. Testosterone is peripherally aromatised to oestrogen resulting in endogenous oestrogen levels which are above the male range but generally lower than the normal female range (276, 277). These oestrogens induce breast development during puberty. In addition, this endogenous oestradiol has positive effects on the skeleton, brain and body habitus in patients with CAIS (269). The patients develop no or scarce pubic and axillary hair. It is generally perceived that in patients with CAIS and intact gonads, spontaneous puberty occurs at the same age as described in the general population although one study reported the start of breast development at a median age of 12–13 years (277). Furthermore, in patients with CAIS, final height is generally above the mean female final height but below the average height of the male population (273, 278, 279).

In individuals with intact gonads, oestrogen treatment can be considered in those with delayed puberty, particularly in individuals with tall stature with the aim to reduce final height. It is known that high dosages of oestrogens used during puberty can be successfully used to reduce final height in girls with constitutional tall stature (280, 281). However, there are no studies reporting the effect of oestrogen treatment (dosage and timing) on the final height in patients with CAIS.

#### Timing of puberty induction, treatment approach and monitoring

**R. 6.2** We recommend to start puberty induction at the age of 11 years in girls with CAIS who underwent gonadectomy. We recommend the same treatment protocols (for pubertal induction in girls with CAIS who have been gonadectomised ) as for other girls (R2.2–2.3, R2.6–2.8, R2.11). (+OOO)

***Rationale***

In girls with CAIS who have been gonadectomised, pubertal induction should start at the age of 11 years in accordance with the current treatment recommendations to mimic normal pubertal development.

Treatment with sex hormones in patients with CAIS is generally only indicated when the gonads have been removed. The timing of puberty should be similar to other indications for pubertal induction in girls.

Patients with CAIS treated with transdermal oestrogens were reported to have better bone health than those treated with oral formulations (269). The risks and benefits of oral vs transdermal E2 are likely to be similar to other cohorts in whom these therapies have been studied more comprehensively, with regard to liver and cardiovascular adverse effects (274). However, the benefits and disadvantages with regard to bone health and sexual function may differ for patients with other diagnoses.

There are still conflicting data concerning the optimal dose of hormonal replacement therapy following puberty induction. Plasma oestradiol levels are positively associated with BMD. Therefore, routine monitoring of oestrogen concentrations by LC-MS/MS and bone density is recommended. Because of the absence of a uterus, the addition of treatment with progestins is generally not required. There is not enough evidence for a beneficial effect on brain, bone or other tissues to recommend the addition of progestin treatment. The use of testosterone rather than oestrogens in an attempt to mimic the hormonal levels of women with CAIS and intact gonads has been studied, particularly with regard to sexual function (274, 282, 283).

An unsolved aspect of the clinical care of young adults with CAIS and intact gonads is that although estrogen treatment is generally not recommended, low E2 plasma levels and BMD can be seen and thus, there may be a need for E2 or testosterone supplementation in these patients (282).

**R 6.3**We recommend treatment monitoring every 3–6 months during puberty induction.

***Rationale***

Regular follow-up with clinical parameters (breast development, height), patient’s satisfaction and bone density. We suggest yearly measurements of FSH and LH as well as E2 levels similarly to (R2).

### Pubertal induction in PAIS

#### In whom to consider puberty induction?

**R. 7.1**We recommend that in all patients with PAIS, evaluation of gender identity should take place before considering puberty induction.

***Rationale***

The clinical picture of PAIS is variable and depends on the residual androgen receptor activity and other modifying partially unknown factors. The genital phenotype ranges from hypospadias to female appearance (284) (Table 1). Furthermore, the degree of virilisation at birth estimated by the EMS has been shown to be a good predictor of virilisation at puberty (175, 258, 259). The external genitalia score (EGS) developed for evaluation in a wider spectrum of genital differences may be used in the future (285).

Careful counselling by a multidisciplinary team is therefore required before any decision about hormonal treatment is taken. An experienced psychologist should evaluate the patients’ gender identity development, preferably from the age of 8 years. Structured psychological assessment of gender should be conducted.

In case there are uncertainties about gender identity, puberty can be delayed by the use of GnRH agonists. Delay of puberty can be helpful in order to gain time to find out which gender will suit the individual best. Psychological follow-up is needed to evaluate gender development over time and keep an eye on the adolescent’s mental health (emotional problems such as social phobia or depression need to be identified and treated). Recognising that gender identity is a non-binary phenomenon can facilitate satisfaction with one’s gender. Detailed information is provided under ‘Psychological Aspects’.

However, pubertal treatment is essential for long-term wellbeing in all patients, and a decision regarding hormonal treatment eventually has to be made.

#### Evaluation of pubertal development

**R. 7.2**We recommend that careful evaluation of patients’ endocrine profile, phenotype and genotype is undertaken and that this information is used to consider the potential for virilisation during puberty (in patients with a male or undecided/binary identity).

***Rationale***

To evaluate the degree of pubertal development in PAIS, we recommend monitoring clinical signs of virilisation, height, full hormonal evaluation, degree of masculinisation at birth (EMS/EGS) and the genetic variant.

At the onset of puberty, the hypothalamic GnRH pulse generator initiates pulsatile GnRH secretion resulting in pulsatile FSH and LH secretion from the pituitary gland. The resulting testosterone secretion from the Leydig cells in the testes fails to exert negative feedback to the hypothalamic-pituitary axis due to insufficient central AR action. However, the increased circulating testosterone is aromatised to oestradiol which exerts normal negative feedback on FSH levels that typically remain within the normal range. Thus, the LH/FSH ratio is typically relatively high in CAIS and PAIS (15, 52). Oestradiol circulates in high-normal concentrations (compared to the male reference range) resulting in gynaecomastia in many boys with PAIS (286, 287). The testicular Sertoli cell markers inhibin B and AMH are within the normal prepubertal male range (48). In the physiological situation, inhibin B increases with pubertal onset, whereas AMH decreases when the Sertoli cells differentiate and start to express AR. With rising intratesticular testosterone levels, AMH secretion is inhibited in a subject with normal AR function, whereas a boy with PAIS continues to have prepubertal levels of AMH due to lack of inhibition. Inhibin B is usually within normal male ranges in boys with PAIS (286).

It is difficult to predict the course of puberty in patients with PAIS. A study analysed EMS at birth in 27 PAIS patients in relation to the pubertal outcome and found that only six of nine patients with EMS < 5 at birth underwent spontaneous male onset of puberty, whereas all 18 patients with EMS ≥ 5 at birth experienced a spontaneous male onset of puberty. In contrast to the clinical findings, the functional analysis of AR variants did not appear to predict pubertal outcome (258). It remains to be seen to which degree the early (peripubertal) changes in reproductive hormones will guide the clinician in the prediction of spontaneous virilisation.

#### Treatment approach and monitoring

**R. 7.3**In children with PAIS identifying as as girls, the general recommendations for pubertal induction in CAIS as formulated under R 6.2–6.3 apply.

***Rationale***

In children with PAIS who have been raised as girls and identify as girls and who have not been gonadectomised, gonadectomy can be considered before or at the very beginning of puberty, to avoid virilisation and gender discomfort/dysphoria in addition to considerations regarding malignancy risk (38, 39, 44, 45, 46, 49). Pubertal induction as recommended for other conditions in which pubertal induction is necessary applies.

**R 7.4**In boys with PAIS, we suggest considering additional testosterone treatment in mid puberty depending on the clinical and biochemical assessment of pubertal development. If clinical signs of hypoandrogenism such as micropenis and gynaecomastia are present, we suggest treating with the addition of testosterone for 6 months and then evaluating the effect. (+OOO)

***Rationale***

The clinical phenotype of individuals with PAIS who identify as males is highly variable from micropenis to severe hypospadias and/or bifid scrotum. It is a theoretical consideration that the inherent androgen insensitivity can be overcome by adding supraphysiological testosterone levels on top of the endogenous testosterone secretion. Anecdotally, patients report a beneficial effect on genital growth and wellbeing, but no randomised trials exist. Transdermal testosterone and long-acting i.m. injections have been tried (287). Clearly, serum testosterone concentrations cannot be used to monitor efficacy, which relies exclusively on clinical improvement and general wellbeing. Biochemically, haematocrit is suggested as a safety parameter. LH has also been suggested as a surrogate marker for optimal dosing of testosterone in males with PAIS.

The majority of males with PAIS develop gynaecomastia (38, 277, 288), which may theoretically either worsen (because of increased E2) or improve (because of lowered LH-induced aromatase activity) with pharmacological testosterone treatment (286). Breast cancer has only been described in a few cases (289). The effect of medical treatment on gynaecomastia is variable and most males decide to undergo mastectomy. Based on the beneficial effects of oestrogen receptor blocking in pubertal gynaecomastia, this treatment modality could also be considered in male patients with PAIS (290). Successful use of tamoxifen, a selective oestrogen receptor blocker, to reduce gynaecomastia was described in two patients with PAIS (291). However, long-term studies are lacking, and whether or not there is a role for oestrogen blockers to prevent gynecomastia remains to be studied.

Other signs of under-virilisation in patients with PAIS raised as boys, such as hypospadias and cryptorchidism, have been surgically corrected during infancy or childhood in most patients.

## Future perspectives

Most of the recommendations in this guideline are based on expert clinical experience and often a long-term clinical experience of treating a large number of individuals. Studies on pubertal induction specifically in individuals with a DSD are scarce. It is clear that further studies are needed. Just to mention a few, issues directly related to hormone treatment with improved formulations and optimal route of administration as well as timing and progression of puberty are needed. The function and importance of the mini-puberty are still largely unknown. The issue of fertility and how the possibility for future fertility can be improved for individuals with different diagnoses are attracting increased attention. Does the treatment with FSH and LH/hCG before the start of testosterone increase the possibility of future fertility in CHH? Studies on optimisation of sex hormone replacement in AIS and how to avoid long-term consequences for these patients are warranted. Animal studies and stem-cell research may open possibilities for the development of novel technologies involving Leydig cell transplantation in male hypogonadism in the future (292).

Diagnostic difficulties to distinguish CHH vs CDGP are still largely unsolved and a clinical recurrent issue. Improved tools for identifying a halt in pubertal development would be of benefit for many patients. Improved psychosocial support for patients is essential.

## Conclusion

Disorders of sexual development comprise a large array of diagnoses and patients with CHH and DSD present different symptoms and needs, especially during puberty. The care for these individuals and their families needs to be individualised and requires the involvement of specialised multidisciplinary teams.

## Supplementary Material

Supplementary Materials

## Declaration of interest

The authors declare that there is no conflict of interest that could be perceived as prejudicing the impartiality of this guidance.

## Funding

This work did not receive any specific grant from any funding agency in the public, commercial, or not-for-profit sector.
