# Supplementary material for: Pubertal induction and transition to adult sex hormone replacement in patients with congenital pituitary or gonadal reproductive hormone deficiency: an Endo-ERN clinical practice guideline
Source: Eur J Endocrinol. 2022 Mar 29;186(6):G9–G49. doi: 10.1530/EJE-22-0073 (PMC9066594; doi:10.1530/EJE-22-0073)
Supplement: Supplementary Materials [file supplementary_material.pdf]

Appendix 1: Clinical questions, eligibility criteria and endpoint definition

Clinical question	Search criteria and key outcome parameters	Number of papers included
Question I: What is the optimal treatment to induce or sustain puberty in males with partial gonadal dysgenesis?	Population: Male patients with partial gonadal dysgenesis<br>Intervention/control: all hormonal treatments used to induce or sustain puberty<br>Outcomes: virilization (Tanner stage, penile length, testicular volume); fertility and sexual function (including spermatogenesis); behavior, psychologic function, and quality of life; bone mineral density and fractures; height, weight, and BMI; adverse outcomes (gynaecomastia, priapism, cardiovascular disease, thrombosis, liver function)	0
Question II: What is the optimal treatment to induce or sustain puberty in males with hypogonadotropic hypogonadism?	Population: Male patients with hypogonadotropic hypogonadism<br>Intervention/control: all hormonal treatments used to induce or sustain puberty<br>Outcomes: virilization (Tanner stage, penile length, testicular volume); fertility and sexual function (including spermatogenesis); behavior, psychologic function, and quality of life; bone mineral density and fractures; height, weight, and BMI; adverse outcomes (gynaecomastia, cardiovascular disease, thrombosis, liver function, non-alcoholic fatty liver disease)	4
Question III: What is the optimal	Population: Female patients with partial gonadal dysgenesis	22 (including 1

treatment to induce or sustain puberty in females with partial gonadal dysgenesis?	Intervention/control: all hormonal treatments used to induce or sustain puberty<br>Outcomes: feminization (Tanner stage, menarche, uterine size); fertility and sexual function; behavior, psychologic function, and quality of life; bone mineral density and fractures; height, weight, and BMI; adverse outcomes (breast cancer, cardiovascular disease, thrombosis, liver function)	study that was also included for question 4)
Question IV: What is the optimal treatment to induce or sustain puberty in females with hypogonadotropic hypogonadism?	Population: Female patients with hypogonadotropic hypogonadism<br>Intervention/control: all hormonal treatments used to induce or sustain puberty<br>Outcomes: feminization (Tanner stage, menarche, uterine size); fertility and sexual function; behavior, psychologic function, and quality of life; bone mineral density and fractures; height, weight, and BMI; adverse outcomes (breast cancer, cardiovascular disease, thrombosis, liver function)	1 (also included for question 3)
Question V: What is the optimal treatment to induce or sustain puberty in patients with complete androgen insensitivity syndrome (CAIS)?	Population: Patients with complete androgen insensitivity syndrome (CAIS)<br>Intervention/control: all hormonal treatments used to induce or sustain puberty<br>Outcomes: feminization (Tanner stage); sexual function; behavior, psychologic function, and quality of life; bone mineral density and fractures; height, weight, and BMI; adverse outcomes (gonadal tumours, cardiovascular disease, thrombosis, liver function)	0
Question VI: What is the optimal treatment to induce or sustain puberty in patients with partial	Population: Patients with partial androgen insensitivity syndrome (PAIS)<br>Intervention/control: all hormonal treatments used to induce or sustain puberty<br>Outcomes: virilization (Tanner stage, penile length, testicular volume); fertility and	0

androgen insensitivity syndrome (PAIS)?	sexual function (including spermatogenesis); behavior, psychologic function, and quality of life; bone mineral density and fractures; height, weight, and BMI; adverse outcomes (gynaecomastia, cardiovascular disease, thrombosis, liver function, non-alcoholic fatty liver disease)	

Appendix 1: Medical treatment to induce or sustain puberty in patients with male partial gonadal dysgenesis.

Table 1: GRADE evidence table

Quality assessment							Number of patients	Effect		Quality	Importance
Number of studies	Design	Risk of bias	Inconsistency	Indirectness	Imprecision	Other considerations		Relative (95% CI)	Absolute		
**Virilization: Tanner stage, penile length, testicular volume**											
0	-	-	-	-	-	None	-	-	-	-	CRITICAL
**Fertility and sexual function (including spermatogenesis)**											
0	-	-	-	-	-	None	-	-	-	-	CRITICAL
**Behaviour, psychologic function, and quality of life**											
0	-	-	-	-	-	None	-	-	-	-	CRITICAL
**Bone mineral density and fractures**											
0	-	-	-	-	-	None	-	-	-	-	CRITICAL
**Height, weight, and BMI**											
0	-	-	-	-	-	None	-	-	-	-	CRITICAL
**Adverse outcomes: gynaecomastia, priapism, cardiovascular disease, thrombosis, liver function**											
0	-	-	-	-	-	None	-	-	-	-	CRITICAL

Appendix 2: Medical treatment to induce or sustain puberty in patients with male hypogonadotropic hypogonadism.

Table 1: GRADE evidence table

Quality assessment							Number of patients	Effect		Quality	Importance
Number of studies	Design	Risk of bias	Inconsistency	Indirectness	Imprecision	Other considerations		Relative (95% CI)	Absolute		
**Virilization: Tanner stage, penile length, testicular volume**											
3	Cohort	Very serious[1]	Not applicable considering different comparisons in each included study	No serious indirectness	Serious[3]	None	Testosterone (n=16)<br><br>hCG (n=43)<br><br>GnRH high dose (n=11)<br><br>GnRH low dose (n=39)	Advancement from Tanner stage II to III took longer, and from Tanner stage IV to V shorter, after testosterone than hCG	11 out of 16 patients reached Tanner stage V after testosterone, 6 out of 12 after hCG<br><br>Penile length: 8.03 cm (SD 1.24) after GnRH, 6.03 cm (SD 1.10) after hCG<br><br>Testicular volume: 2-8 ml after testosterone, 6.01-14.0 ml after hCG, 5-25 ml after high dose GnRH, 6-20 ml after low dose GnRH	⊕OOO VERY LOW	CRITICAL
**Fertility and sexual function (including spermatogenesis)**											
2	Cohort	Very serious[1]	Not applicable considering different comparisons in each included study	No serious indirectness	Serious[3]	None	hCG (n=21)<br><br>GnRH high dose (n=8)<br><br>GnRH low dose (n=36)	-	hCG: no spermatogenesis<br><br>GnRH high dose: spermatogenesis in 6 patients (0.1-28 *10^6/ml)<br><br>GnRH low dose: spermatogenesis in 21 patients (0.1-45 *10^6/ml)	⊕OOO VERY LOW	CRITICAL

**Behaviour, psychologic function, and quality of life**											
0	-	-	-	-	-	None	-	-	-	-	CRITICAL
**Bone mineral density and fractures**											
1	Randomized trial	Very serious[2]	Not applicable with one single study only	No serious indirectness	Very serious[3]	None	Testosterone (n=6)<br><br>No treatment (n=6)	-	Increase in bone mineral density in all patients with testosterone, no significant increase in bone mineral density in patients without treatment	⊕OOO VERY LOW	CRITICAL
**Height, weight, and BMI**											
2	Cohort	Very serious[1]	Not applicable considering different comparisons in each included study	No serious indirectness	Serious[3]	None	Testosterone (n=16)<br><br>hCG (n=43)<br><br>GnRH low dose (n=12)	-	Height: 174.6 cm (SD 5.7) after testosterone, 159.15-175.1 cm (SD 6.1-17.03) after hCG, 162.85 cm (SD 12.63) after GnRH<br><br>Weight: 54.84 kg (SD 13.38) after hCG, 57.02 kg (SD 11.85) after GnRH<br><br>BMI: 21.83 kg/m2 (SD 6.52) after hCG, 20.94 kg/m2 (SD 4.42) after GnRH	⊕OOO VERY LOW	CRITICAL
**Adverse outcomes: gynaecomastia, cardiovascular disease, thrombosis, liver function, non-alcoholic fatty liver disease**											
0	-	-	-	-	-	None	-	-	-	-	CRITICAL

[1]Incomplete follow-up, missing data, lack of adjustment in statistical analysis to control for confounding

[2]Lack of blinding, randomization and allocation procedure not reported

[3]Small sample size

Table 2: Details of included studies

Reference, study design	Treatment and number of patients per treatment; other treatments used; age at start of therapy; treatment duration	Relevant outcomes per treatment
Arisaka *et al.* 1995<br>Metabolism<br>Randomized trial	Testosterone enanthate 125 mg intramuscular (n=6), no treatment (n=6); growth hormone; median 18 years (range 15-21); 12 months	Increase in **bone mineral density** in all patients with testosterone, no significant increase in bone mineral density in patients without treatment
Bistritzer *et al.* 1989<br>Fertil Steril<br>Cohort	Monthly long-acting testosterone intramuscular (20 mg proprionate, 80 mg heptanoate, 150 mg undecylate) (n=16), weekly intramuscular 5000 IU hCG (n=22); none described; range 14-21 years; 2.5 years	Advancement from **Tanner stage** II to III took longer, and from Tanner stage IV to V shorter, in the testosterone than in the hCG group; 11 out of 16 patients reached Tanner stage V in the testosterone group and 6 out of 12 in the hCG group; mean final **testicular volume** was 4.3 ml (SD 1.8, range 2-8) for the testosterone group and 14.0 ml (SD 2.0, range 8-25) for the hCG group; mean final **height** was 174.6 cm (SD 5.7) for the testosterone group and 175.1 cm (SD 6.1) for the hCG group
Delemarre-Van de Waal 1993<br>Clin Endocrinol<br>Cohort	GnRH starting dose 20 µg/90 minutes (n=11), GnRH starting dose 2-10 µg/90 minutes (n=27); part of the patients previously used androgens, hMG, and/or hCG, part had orchidopexia; mean 18.9 years (range 13.4-26.0); 22-167 weeks (excluding 2 patients on a long term fixed-dose protocol)	**Testicular volume** was 5-25 ml in the high dose group, and 6-20 ml in the low dose group; **spermatogenesis** was seen in 6 out of 8 patients (0.1-28 *10^6/ml) in the high dose group, and in 20 out of 24 patients (0.1-45 *10^6/ml) in the low dose group
Gong *et al.* 2015<br>J Clin Endocrinol Metab<br>Cohort	GnRH 8-10 µg/90 minutes subcutaneous (n=12), hCG 2000-7000 IU intramuscular per week (n=21); none described; range 10-16 years; 12 months	**Testicular volume** 13.09 mL (SD 3.82) in GnRH group and 6.01 mL (SD 1.33) in hCG group; **penile length** 8.03 cm (SD 1.24) in GnRH group and 6.03 cm (SD 1.10) in hCG group; **spermatogenesis**: nocturnal emission in 1 patient in GnRH group and no patients in hCG group; **height** 162.85 cm (SD 12.63) in GnRH group and 159.15 cm (SD 17.03) in hCG group; **weight** 57.02 kg (SD 11.85) in GnRH group and 54.84 kg (SD 13.38) in hCG group; **BMI** 20.94 kg/m2 (SD 4.42) in GnRH group and 21.83 kg/m2 (SD 6.52) in hCG group

Appendix 3: Medical treatment to induce or sustain puberty in patients with female partial gonadal dysgenesis.

Table 1: GRADE evidence table

**Quality assessment**							**Number of patients**	**Effect**		**Quality**	**Importance**
**Number of studies**	**Design**	**Risk of bias**	**Inconsistency**	**Indirectness**	**Imprecision**	**Other considerations**		**Relative (95% CI)**	**Absolute**		
**Feminization: Tanner stage, menarche, uterine size**											
6<br><br>5	Randomized trial<br><br>Cohort	Very serious[1]	Very serious[2]	No serious indirectness	Serious[3]	None	In total: 322	Risk ratio of reaching Tanner stage B3 during study of oral conjugated (n=14) versus transdermal 17β estradiol (n=11): 0.88 (95% CI: 0.62-1.24)<br><br>Tanner stage: individual studies show no clear advantage for high or low dose, individualized or fixed dose, estrogen or no estrogen, early or late start of treatment<br><br>Menarche: individual studies show no clear advantage for estrogen or no estrogen, oral or transdermal estrogen, early or late start of treatment<br><br>Uterine size: individual studies show that longer duration and higher dose of estrogen results in a larger uterus, no clear advantage for oral or transdermal estrogen, early or late start	-	⊕OOO VERY LOW	CRITICAL

								of treatment			
**Fertility and sexual function**											
0	-	-	-	-	-	None	-	-	-	-	CRITICAL
**Behaviour, psychologic function, and quality of life**											
0	-	-	-	-	-	None	-	-	-	-	CRITICAL
**Bone mineral density and fractures**											
2<br><br>4	Randomized trial<br><br>Cohort	Very serious[1]	Very serious[2]	No serious indirectness	Serious[3]	None	In total: 806	Bone mineral density: individual studies show that transdermal estrogens lead to a greater increase in bone mineral density than oral estrogens, no clear advantage for estrogens versus no estrogens, low or high dose, early or late start of treatment	-	⊕OOO VERY LOW	CRITICAL
**Height, weight, and BMI**											
8<br><br>1<br><br><br>6	Randomized trial<br><br>Non-randomized trial<br><br>Cohort	Very serious[1]	Very serious[2]	No serious indirectness	Serious for some comparisons[3]<br><br>No serious imprecision for comparisons in meta-analysis	None	In total: 589	Difference in height for estrogen (n=61) versus no estrogen (n=69): -2.9 cm (95% CI: -4.9, -0.8)<br><br>Difference in height for early (n=100) versus late treatment (n=110): -1.0 cm (95% CI: -4.0, 1.9)<br><br>Height: individual studies show a larger height after a higher dose, no clear advantage for oral or transdermal estrogen<br><br>Weight: individual studies show no clear advantage for	-	⊕OOO VERY LOW	CRITICAL

								estrogen versus no estrogen, oral or transdermal estrogen<br><br>Difference in BMI for early (n=31) versus late treatment (n=44):<br>-0.9 kg/m2 (95% CI: -2.7, 0.9)<br><br>BMI: individual studies show no clear advantage for estrogen versus no estrogen, individualized or fixed dose			
**Adverse outcomes: breast cancer, cardiovascular disease, thrombosis, liver function**											
2	Cohort	Very serious[1]	Not applicable with 1 study per comparison	No serious indirectness	No serious imprecision	None	In total: 724	Liver function: individual studies show no clear difference between estrogens or no estrogens, age at start of treatment	-	⊕OOO VERY LOW	CRITICAL

[1]Trials: lack of blinding, randomization and allocation procedure not reported for all trials, loss to follow-up; cohort studies: inclusion method for patients not reported for all studies, missing data, no adjustment for confounding variables

[2]Many different comparisons with conflicting results

[3]Small sample sizes per comparison (high versus low dose, individualized versus fixed dose, estrogen versus no estrogen, oral versus transdermal, early versus late start of treatment)

Table 2: Details of included studies

Reference, study design	Population; treatment and number of patients per treatment; other treatments used; age at start of therapy; treatment duration	Relevant outcomes per treatment
Cakir *et al.* 2015<br>J Pediatr Endocrinol Metab<br>Cohort	Turner syndrome; oral estradiol hemihydrate, mean dose 0.030 mg/kg/day (±0.011, range 0.014–0.045) (n=6), transdermal estradiol, mean dose 0.14 µg/kg/day (±0.04, range 0.08–0.18) (n=7); growth hormone, progesterone after menarche; oral: mean 14.2 years (range 12.55-17.33), transdermal: mean 13.77 years (range 11.13-15.62); 1.01-2.39 years	Time in years to each **Tanner breast stage** was longer after oral than after transdermal estradiol (oral: stage 2: 0.46 ±0.207, range 0.17–0.75, stage 3: 0.457 ±0.36, range 0.25–1, stage 4: 0.99 ±0.44, range 0.75–1.66, stage 5 (n=2): 0.83 ±0.46, range 0.5–1.16, transdermal: stage 2: 0.396 ±1.76, range 0.25–0.71, stage 3: 0.32 ±0.067, range 0.25–0.43, stage 4: 0.64 ±0.52, 0.27–1.01); all 6 with oral estradiol had **menarche** during study period versus none with transdermal estradiol; Turner syndrome **height** SD score oral estradiol: -0.68 to 1.64, transdermal estradiol: -0.9 to 2.29
Cameron-Pimblett *et al.* 2019<br>J Clin Endocrinol Metab<br>Cohort	Turner syndrome; continuous comparisons of various treatment strategies using estrogen, majority ethinyl estradiol starting dose 2 µg/day (n=624); not described; median 14 years (95% CI: 5-23.4); not described	**Bone density** T-scores of the hip and spine showed a negative correlation with estrogen start age (r=20.20 and r=20.22 respectively); no correlation between starting age of estrogens and **liver enzymes**
Chernausek *et al.* 2000<br>J Clin Endocrinol Metab<br>Randomized trial	Turner syndrome; conjugated estrogen 0.3-0.625 mg/day starting age 12 years (n=26), same treatment starting age 15 years (n=29); growth hormone, one year after start of estrogens: progesterone; early group mean 12.3 years (SD 0.3, range 11.9-13.2), late group mean 15.0 years (SD 0.5, range 13.6-16.0); not described	Progression through **Tanner breast stages** not different between both groups; **height** early group 147.0 cm (SD 6.1, range 135.0-155.4), late group 150.4 cm (SD 6.0, range 141.1-162.0)
Demetriou *et al.* 1984<br>Obstet Gynecol<br>Cohort	Turner syndrome; early estrogen treatment, most conjugated estrogen, dose 0.3-0.625 mg/day (n=19), late conjugated estrogen treatment, dose 0.3-1.25 mg/day (n=18); progesterone added after average 1.9 years of treatment; early group: mean 14.3 years (SD 1.0, range 11.4-15.5); late group: mean 17.2 years (SD 1.2, range 15.6-19.2); early group: 4.4 years (SD 1.4), late group: 2.1 years (SD 1.6)	**Height** early group: 142.1 cm (SD 4.2, range 134.6-148.6), late group: 143.0 cm (SD 5.4, range 130.8-150.5)
Elsedfy *et al.* 2012<br>J Pediatr Endocrinol Metab	Turner syndrome; continuous comparisons of various treatment strategies using estrogen, mean 17β-estradiol equivalent dose 1.19 mg/day (SD 0.82, range 0.16-2.78) (n=23); part used growth	**Uterine volume** was significantly related to years of estrogen use (longer use = larger uterus) and mean estrogen dose (higher dose = larger uterus), there were no significant

Cohort	hormone and/or progesterone; mean 17.52 years (SD 1.95, range 14.69-22.3); 2.86 years (SD 2.35, range 0.33-7.23)	differences for different types of estrogen, or age at first estrogen exposure
Ferrández *et al.* 1989<br>Acta Paediatr Scand<br>Trial	Turner syndrome; ethinyl estradiol 100 ng/kg/day + growth hormone (n=15), growth hormone (n=18), growth hormone + oxandrolone (n=15); none; estradiol group: mean 12.0 years (SD 1.5), growth hormone group: mean 7.1 years (SD 3.0), oxandrolone group: mean 9.5 years (SD 2.1); 6 months	**Height** SDS: estrogen group -2.67 (SD 1.23), growth hormone group -2.38 (SD 0.62), oxandrolone group 2.54 (SD 1.27); **weight** SDS: estrogen group -1.12 (SD 0.96), growth hormone group -1.84 (SD 1.20), oxandrolone group 1.28 (SD 1.38)
Folsom *et al.* 2017<br>Endocr Pract<br>Cohort	Turner syndrome; continuous comparisons of various treatment strategies using estrogens including transdermal ethinyl estradiol, oral conjugated estrogen, and oral ethinyl estradiol, initial dose range from 0.00175 mg transdermal to 0.625 mg oral (n=43); progesterone; mean 13.9 years (SD 1.9, range 11.3-19); 2.4 years (SD 1.1, range 0.5-4.74)	Younger age at estrogen initiation correlated with a longer time to **menarche**, there was no correlation with estrogen route or dose; a greater proportion of girls who bled on estrogen alone (as opposed to also progesterone) were treated with transdermal estrogen
Gault *et al.* 2019<br>Arch Dis Child<br>Randomized controlled trial	Turner syndrome; estrogen at 12 years, dose 2-10 µg/day (n=17), estrogen at 14 years, same dose protocol (n=15); growth hormone, progesterone from age 15; 12 or 14 years; 3 years	**Height**: early group 148.1 cm (SD 7.2), late group 152.4 cm (SD 6.6)
Golden *et al.* 2002<br>J Pediatr Adolesc Gynecol<br>Cohort	Hypogonadism due to anorexia nervosa; ethinyl estradiol 20-35 µg + cyclic progesterone (n=18), no estrogen/progestin (n=25); standard treatment for anorexia nervosa; mean 16.8 years (SD 2.3); 3 years	**Bone mineral density**: lumber spine 0.834 g/cm2 (SD 0.07), femoral neck 0.694 g/cm2 (SD 0.11) for the estrogen group, and lumber spine 0.819 g/cm2 (SD 0.09), femoral neck g/cm2 0.723 (SD 0.09) for the no estrogen group; **weight** estrogen group 48.8 kg (SD 5.6), no estrogen group 47.5 kg (SD 6.1)
Hasegawa *et al.* 2017<br>Endocr J<br>Cohort	Turner syndrome; early estrogens, starting dose 1-5 ng/kg ethinyl estradiol, later switch to conjugated estrogen (n=17), late estrogens, initial dose 0.3125 mg/week of conjugated estrogens (n=30); growth hormone, progesterone; early group mean 11.6 years (range 9.8-13.7), late group mean 15.4 years (range 12.2-18.7); early group mean 4.2 year (SD 0.8)	Volumetric **bone mineral density**: early group 0.262 g/cm3 (SD 0.027), late group 0.262 g/cm3 (SD 0.033); **height**: early group 152.4 cm (SD 3.4), late group 148.5 cm (SD 3.0); **BMI**: early group 21.8 kg/m2 (SD 4.5), late group 22.4 kg/m2 (SD 2.9)
Kim *et al.* 2012<br>Gynecol Endocrinol<br>Cohort	Turner syndrome; estradiol valerate initial dose 0.5 mg/day (n=9), estrogen initial dose 1 mg/day (n=10); growth hormone, progestin was added later, calcium + vitamin D; low dose group median 14.6 years (range 13.0-17.4), high dose group median 15.94 years (range 14.3-17.6); at least 1 year	**Tanner breast stage** 2 was reached by all patients; **uterine size**: length 4.78 cm (SD 0.68) and anterior-posterior fundal diameter 1.18 cm (SD 0.37) for the low dose group, length 5.85 cm (SD 0.96) and anterior-posterior fundal diameter 1.81 cm (SD 0.54) for the high dose group; **bone mineral**

		**density** lumbar 0.75 g/m2 (SD 0.07), femur neck 0.67 g/m2 (SD 0.07) for low dose group, lumbar 0.78 g/m2 (SD 0.10), femur neck 0.67 g/m2 (SD 0.09) for high dose group; **height** low dose group 146.42 cm (SD 3.28), high dose group 151.81 cm (SD 4.89)
Labarta *et al.* 2012<br>Eur J Endocrinol<br>Randomized trial	Turner syndrome; individualized estrogen dose 5-15 µg/kg (n=22), fixed estrogen dose 0.2-0.5 mg (n=23); growth hormone; individualized dose group mean 14.3 years (SD 1.4), fixed dose group mean 13.4 years (SD 1.0); 2 years	Time to **Tanner stage** B4: 818 days, percentage reaching B4: 42.0% (95% CI: 22.0-63.0), percentage reaching P4: 71.0% (95% CI: 49.0-87.0) for individualized dose group, time to B4: 733 days, percentage reaching B4: 65.0% (95% CI: 46.0-85.0), percentage reaching P4: 87.0% (95% CI: 66.0-97.0) for fixed dose group; **BMI** SD score 1.27 (SD 0.24) for individualized dose group, and for fixed dose group SD score 1.38 (SD 0.26)
Misra *et al.* 2011<br>J Bone Miner Res<br>Randomized controlled trial	Hypogonadism due to anorexia nervosa; transdermal 17β-estradiol 200 mg/week or oral ethinyl estradiol, starting dose 3.75 µg/day (n=31), placebo (n=30); progesterone from age 15 in estrogen group, calcium + vitamin D; mean 16.5 years (SE 0.2, range 12-18); 18 months	Significantly greater increase in **bone mineral density** Z-score at spine and hip in estrogen than placebo group; no difference in change in **weight** or **BMI**
Nabhan *et al.* 2009<br>J Clin Endocrinol Metab<br>Randomized trial	Turner syndrome; conjugated oral estrogen, starting dose 0.3 mg/day (n=6), transdermal estrogen, starting dose 0.05 mg/week (n=6); growth hormone, progesterone after first bleeding; mean 14.0, SD 1.7 (range 11.3-17.1) years; 12 months	Number to reach **Tanner breast stage** III-IV: both groups 5 out of 6; number with breakthrough **bleeding**: oral group 1 out of 6, transdermal group 4 out of 6; oral group had a smaller increase in **uterine size** than transdermal group: length change oral 1.98 cm (SD 0.39), transdermal 4.13 cm (SD 0.39), and volume change oral 4.0 ml (SD 4.4) and transdermal 22.2 ml (SD 4.4), number with mature uterus after 12 months (length ≥6.50cm): oral 0 out of 6, transdermal 4 out of 6; oral group had a smaller increase in **bone mineral density** than transdermal group: change oral 0.06 g/cm2 (SD 0.01), transdermal 0.12 g/cm2 (SD 0.01), and Z-score change oral 0.7 (SD 0.1), transdermal 0.3 (SD 0.1)
Naeraa *et al.* 1994<br>Eur J Pediatr<br>Cohort	Turner syndrome; oral micronized estrogen, starting dose 0.01 mg/kg/day (n=8), same estrogen protocol + growth hormone (n=18), only growth hormone (n=13); none; estrogen only group mean 11.4 years (SD 1.4, range 9.6-13.2), estrogen + growth hormone group	**Tanner breast stage** progression: estrogen group after 1 year breast stage was unchanged in 1 girl, progressed 1 stage in 5 girls and progressed 3 stages in 2 girls, after 2 years unchanged in 2 girls, another stage progression in 6 girls,

	mean 14.4 years (SD 1.7, range 11.8-18.1), only growth hormone group mean 9.4 years (SD 1.2, range 7.6-11.7); 2 years	estrogen + growth hormone group after 1 year breast stage progressed 1 stage in 9 girls, 2 stages in 6 girls, unchanged in 1 girl, after 2 years 8 girls unchanged, the others progressed 1 stage, only growth hormone group after 1 year breast stage progressed 1 stage in 1 girl, after 2 years 8 girls unchanged, others progressed 1 stage; there were irregular vaginal **bleedings** in 2 girls in the estrogen + growth hormone group
Nilsson *et al.* 1996<br>J Clin Endocrinol Metab<br>Randomized trial	Turner syndrome; ethinyl estradiol 100 ng/kg/day (n=15), no estrogens (n=16); growth hormone + oxandrolone; mean 12.2 years (range 9-16); unclear	**Height** estrogen group 151.1 cm (SD 4.6), no estrogen group 154.2 cm (SD 6.6)
Perry *et al.* 2014<br>Horm Res Paediatr<br>Randomized controlled trial	Turner syndrome; estrogen at 12 years, dose 2-10 µg/day (n=17), estrogen at 14 years, same dose protocol (n=25); growth hormone, progesterone from age 15; 12 or 14 years; 3 years	Age at **Tanner breast stage** early group stage 1: 12.5 years (SD 0.9), stage 2: 12.9 years (SD 1.1), stage 3: 13.7 years (SD 1.2), stage 4: 14.5 years (SD 1.1), late group stage 1: 13.3 years (SD 1.7), stage 2: 14.2 years (SD 1.5), stage 3: 15.1 years (SD 1.5), stage 4: 15.8 years (SD 1.5); age at **menarche** late group 15.9 years (SD 1.6), no data on early group
Quigley *et al.* 2002<br>J Clin Endocrinol Metab<br>Randomized controlled trial	Turner syndrome; ethinyl estradiol mean 100 ng/kg/day (n=46), placebo (n=53); growth hormone high or low dose; mean 9.7 years (SD 2.8); 18 months	**Height** estrogen + low dose growth hormone 145.1 cm (SD 5.4), estrogen + high dose growth hormone 149.1 cm (SD 6.0), placebo + low dose growth hormone 149.9 cm (SD 6.0), placebo + high dose growth hormone 150.4 cm (SD 6.0)
Rosenfield *et al.* 2005<br>J Clin Endocrinol Metab<br>Randomized trial	Turner syndrome; estradiol cypionate 0.2-3.0 mg/months intramuscular from 12-13 years (n=7), same estrogen treatment protocol from 14-15 years (n=4); growth hormone, progesterone after 4 years or after menarche; 12-13 years or 14-15 years; at least 4 years	**Menarche** occurred in 5 out of 7 patients before study end in the early group (age range 13.25-15.40 years), and in 1 patient in the late group (age 17.8 years); **height** mean 154.0 cm (range 148.6-162.0) in the early group, mean 152.9 cm (range 151.2-155.5) in the late group
Ruszala *et al.* 2017<br>J Endocrinol Invest<br>Cohort	Turner syndrome; 17β-estradiol, 62.5 µg/day before age 12, followed by standard pubertal induction regimen after age 12 (n=14), standard pubertal induction regimen after age 12 (n=14); growth hormone; early group mean 10.5 years (SD 0.95), late group mean 14 years (SD 1.96); 3 years	**Height** early group 146.8 cm (SD 9.8), late group 152.2 cm (SD 7.2); **BMI** early group 20.0 kg/m2 (SD 3.5), late group 21.8 kg/m2 (SD 5.6)
Shah *et al.* 2014<br>Int J Pediatr Endocrinol<br>Randomized trial	Hypogonadism due to Turner syndrome, or primary or secondary ovarian failure; oral 17β estradiol 0.25-1 mg/day (n=7), transdermal 17β estradiol 0.0125-0.05 mg/day (n=5), oral conjugated estrogen	Number to reach **Tanner breast stage** 3: oral and transdermal estradiol groups all patients, oral conjugated estrogen group 6 out of 8 patients; **height** oral estradiol

	0.15-0.625 mg/day (n=8); part growth hormone; oral estradiol group mean 14.5 years (SE 0.6), transdermal estradiol group mean 14.2 years (SE 0.5), oral conjugated estrogen group mean 13.8 years (SE 0.4); 18 months	group 147.6 cm (SE 3.9), transdermal estradiol group 148.6 cm (SE 3.5), oral conjugated estrogen group 153.2 cm (SE 2.9); **weight** oral estradiol group 43.6 kg (SE 4), transdermal estradiol group 40.6 kg (SE 3.1), oral conjugated estrogen group 53.8 kg (SE 10.1)
Wójcik *et al.* 2019<br>J Clin Res Pediatr Endocrinol<br>Cohort	Turner syndrome; various treatment strategies using estrogens (n=44), no estrogens (n=56); growth hormone; range 4-16; mean 4.31 years (SD 0.82)	**Liver function** in estrogen group: elevated liver function tests in 16 patients (36%), increased AST in 5 patients (11%), increased ALT in 11 patients (25%), mean AST 44.2 IU/L, mean ALT 29.9 IU/L, none developed overt liver disease, and in no estrogen group: elevated liver function tests in 18 patients (32%), increased AST in 10 patients (18%), increased ALT in 9 patients (16%), mean AST 42.7 IU/L, mean ALT 27.5 IU/L, none developed overt liver disease

Appendix 4: Medical treatment to induce or sustain puberty in patients with female hypogonadotropic hypogonadism.

Table 1: GRADE evidence table

**Quality assessment**							**Number of patients**	**Effect**		**Quality**	**Importance**
**Number of studies**	**Design**	**Risk of bias**	**Inconsistency**	**Indirectness**	**Imprecision**	**Other considerations**		**Relative (95% CI)**	**Absolute**		
**Feminization: Tanner stage, menarche, uterine size**											
1	Randomized trial[1]	Very serious[2]	Not applicable with one single study only	Serious[3]	Very serious[2]	None	Oral 17β estradiol (n=7)<br><br>Transdermal 17β estradiol (n=5)<br><br>Oral conjugated equine estrogen (n=8)	-	Oral 17β estradiol: all patients reached Tanner stage B3<br><br>Transdermal 17β estradiol: all patients reached Tanner stage B3<br><br>Oral conjugated equine estrogen: 6 out of 8 patients reached Tanner stage B3	⊕OOO VERY LOW	CRITICAL
**Fertility and sexual function**											
0	-	-	-	-	-	None	-	-	-	-	CRITICAL
**Behaviour, psychologic function, and quality of life**											
0	-	-	-	-	-	None	-	-	-	-	CRITICAL
**Bone mineral density and fractures**											
0	-	-	-	-	-	None	-	-	-	-	CRITICAL
**Height, weight, and BMI**											

1	Randomized trial[1]	Very serious[2]	Not applicable with one single study only	Serious[3]	Very serious[2]	None	Oral 17β estradiol (n=7)<br><br>Transdermal 17β estradiol (n=5)<br><br>Oral conjugated equine estrogen (n=8)	-	Oral 17β estradiol: height 147.6 cm (SE 3.9), weight 43.6 kg (SE 4)<br><br>Transdermal 17β estradiol: height 148.6 cm (SE 3.5), weight 40.6 kg (SE 3.1)<br><br>Oral conjugated equine estrogen: height 153.2 cm (SE 2.9), weight 53.8 kg (SE 10.1)	⊕OOO VERY LOW	CRITICAL
**Adverse outcomes: breast cancer, cardiovascular disease, thrombosis, liver function**											
0	-	-	-	-	-	None	-	-	-	-	CRITICAL

[1]Shah *et al.* Int J Pediatr Endocrinol 2014, 2014: 12.

[2]Very small sample size per group, from which some patients were lost to follow-up and some were excluded from analysis due to poor adherence.

[3]Study included both patients with hypogonadotropic hypogonadism and partial gonadal dysgenesis without presenting separate outcomes.

Appendix 5: Medical treatment to induce or sustain puberty in patients with complete androgen insensitivity syndrome.

Table 1: GRADE evidence table

Quality assessment							Number of patients	Effect		Quality	Importance
Number of studies	Design	Risk of bias	Inconsistency	Indirectness	Imprecision	Other considerations		Relative (95% CI)	Absolute		
**Feminization: Tanner stage**											
0	-	-	-	-	-	None	-	-	-	-	CRITICAL
**Sexual function**											
0	-	-	-	-	-	None	-	-	-	-	CRITICAL
**Behaviour, psychologic function, and quality of life**											
0	-	-	-	-	-	None	-	-	-	-	CRITICAL
**Bone mineral density and fractures**											
0	-	-	-	-	-	None	-	-	-	-	CRITICAL
**Height, weight, and BMI**											
0	-	-	-	-	-	None	-	-	-	-	CRITICAL
**Adverse outcomes: gonadal tumours, cardiovascular disease, thrombosis, liver function**											
0	-	-	-	-	-	None	-	-	-	-	CRITICAL

Appendix 6: Medical treatment to induce or sustain puberty in patients with partial androgen insensitivity syndrome.

Table 1: GRADE evidence table

Quality assessment							Number of patients	Effect		Quality	Importance
**Number of studies**	**Design**	**Risk of bias**	**Inconsistency**	**Indirectness**	**Imprecision**	**Other considerations**		**Relative (95% CI)**	**Absolute**		
**Virilization: Tanner stage, penile length, testicular volume**											
0	-	-	-	-	-	None	-	-	-	-	CRITICAL
**Fertility and sexual function (including spermatogenesis)**											
0	-	-	-	-	-	None	-	-	-	-	CRITICAL
**Behaviour, psychologic function, and quality of life**											
0	-	-	-	-	-	None	-	-	-	-	CRITICAL
**Bone mineral density and fractures**											
0	-	-	-	-	-	None	-	-	-	-	CRITICAL
**Height, weight, and BMI**											
0	-	-	-	-	-	None	-	-	-	-	CRITICAL
**Adverse outcomes: gynaecomastia, cardiovascular disease, thrombosis, liver function, non-alcoholic fatty liver disease**											
0	-	-	-	-	-	None	-	-	-	-	CRITICAL
